# Genetically-engineered *Salmonella typhimurium* expressing FGF21 promotes neurological recovery in ischemic stroke via FGFR1/AMPK/mTOR pathway

**DOI:** 10.1186/s12974-025-03498-0

**Published:** 2025-06-28

**Authors:** Dongchen Xu, Min Wen, Bingwa Lebohang Anesu, Xijun Chen, Yuhao Chen, Wenqi Qian, Chenguang Yang, Jin Hai Zheng, Yinan Zhou, Haoqi Ni, Kunlin Jin, Qichuan Zhuge, Su Yang

**Affiliations:** 1 Zhejiang - US Joint Laboratory for Aging and Neurological Disease Research, The First Affiliated Hospital of Wenzhou Medical University, Wenzhou, 325000 China; 2 Department of Neurosurgery, The First Affiliated Hospital of Wenzhou Medical University, Wenzhou, 325000 China; 3 Department of Neurosurgery, Guangzhou First People’s Hospital, School of Medicine, South China University of Technology, Guangzhou, 510000 China; 4 Institute of Hypoxia Medicine, School of Basic Medical Sciences, Wenzhou Medical University, Wenzhou, 325035 China; 5 School of Biomedical Sciences, Hunan University, Changsha, 410082 China; 6 Department of Pharmacology and Neuroscience, University of North Texas Health Science Center, Fort Worth, TX 76107 USA

**Keywords:** Ischemic stroke, *Salmonella typhimurium*, FGF21, Neurologic deficit, FGFR1/AMPK/mTOR pathway, Hepatorenal histopathology

## Abstract

**Background:**

Ischemic stroke (IS) remains a leading cause of mortality and disability, with limited therapeutic options due to poor drug delivery to ischemic lesions. To address this challenge, an engineered *Salmonella* based therapeutic method for targeted drug delivery and long-term treatment is herein designed to mitigate ischemic damage.

**Methods:**

We engineered an attenuated luminescent *Salmonella typhimurium* (*S.t* -ΔpG) strain with an L-arabinose-inducible pBAD system to secrete bioactive FGF21. C57BL/6 mice were used to to measure neuron apoptosis and the activity of immune cells following IS induction plus *S.t*-ΔpG injection. Bioluminescence imaging was applied for bacterial colonization. ELISA and glucose uptake assays were performed to detect FGF21 secretion and the bioactivity. Neurological tests, TTC staining, and TUNEL labeling were used to assess the therapeutic effects of barterially secreted FGF21. Immunofluorescence assay of FGF21/FGFR1 dominant pathway was explored to investigate neuroprotective mechanism, while IBA-1 staining, CD3/CD68 immunostaining, cytokine profiling, and hepatorenal histopathology were detected to evaluate biosecurity.

**Results:**

*S.t*-ΔpG^FGF21^ selectively colonized peri-infarct regions and secreted functional FGF21, reducing neurologic deficits (48%) and infarct volume (46%) versus controls (*p* < 0.01). Mechanistically, immunofluorescence demonstrated that bacterially secreted FGF21 activated neuronal FGFR1/AMPK/mTOR pathway to enhance autophagy, whereas autophagy inhibition abolished its neuroprotection. Further, bacterial exclusion from neuron was validated via MAP2/NeuN plus *Salmonella* co-staining in primary neuron cells and brain tissue. Critically, CD3/CD68 immunostaining, serum cytokine profiling, and hepatorenal histopathology confirmed the long-term biosafety of this approach.

**Conclusion:**

Our study presents a novel, *Salmonella* - based platform for targeted and sustained FGF21 delivery, offering a promising therapeutic strategy for ischemic stroke with robust efficacy and minimal systemic toxicity.

**Supplementary Information:**

The online version contains supplementary material available at 10.1186/s12974-025-03498-0.

## Introduction

Ischemic stroke (IS) is a life-threatening cerebrovascular disease with high rates of mortality and morbidity, which emerges complex pathological cascades that culminate in irreversible neurological deficits [1–3]. While current therapies focus on reperfusion (e.g., thrombolysis) and penumbra salvage [4, 5], secondary brain injury (SBI) perpetuates neuronal death and limits functional recovery [6, 7], underscoring the urgent need for neuroprotective strategies.

Currently, targeted therapies are urgently needed to address neural cell death caused by SBI. However, the blood-brain barrier (BBB), a specialized capillary barrier in the brain, presents huge obstacle to the treatment of ischemia stroke as it impedes most drugs and delivery systems from entering the brain while maintaining homeostasis and protecting the brain from toxic substances [8, 9]. Owing to the relatively anaerobic environment of cerebral infarct foci, biological therapies, such as in situ viral and bacterial therapies, have been exploited for BBB transportation and hold promise as alternative effective intervention strategies [10–12].

Traditional delivery methods for neuropretective durgs, like viral vectors and nanoparticles, have their own drawbacks. Viral vectors, although highly efficient in gene delivery, may trigger immune responses, leading to potential safety concerns [13]. Moreover, the risk of insertional mutagenesis is also a major issue. Nanoparticles, on the other hand, often have limited loading capacity and may encounter challenges in achieving targeted delivery to the ischemic brain tissue [14–16].

Recently, the use of bacteria as delivery vectors has emerged as an alternative strategy. *Salmonella*, in particular, has attracted attention due to its natural ability to target ischemic tissues [17]. Recently *Salmonella typhimurium* (*S. t*) has been utilized in the development of treatment for brain diseases, including brain tumor and antibiotic research [18, 19]. Our previous study has demonstrated that attenuated *Salmonella typhimurium* (*S.t*-ΔpG) could cross the BBB to target orthotopic glioma in mice models while eliciting low toxicity to the host [20]. It is able to precisely target hypoxic regions in solid tumors and multiply therein. This property can be used to target delivery of protein drugs to infarcted brain tissue, highlighting its applications of synthetic biology methods in hypoxic and necrotic regions. In this regard, we have fabricated luminescence manipulator (luciferase gene; *lux*) labeled attenuated *Salmonella* (*S.t*-ΔpG^lux^) based on genetic engineering methods.

Fibroblast growth factor 21 (FGF21) is a multifunctional metabolic stress hormone and predominately expressed in liver. Recent studies highlight its neuroprotective, anti-inflammatory, and pro-repair effects of FGF21 in ischemic brain injury, making it a promising therapeutic candidate for stroke recovery [21–23]. Multiple evidences show FGF21 could reduces neuronal apoptosis, repairs the damaged blood-brain barrier, reduces the size of infarct foci and improves neurological deficits [24–26]. Others have reported that FGF21 presents anti-inflammatory properties by modulating microglia and macrophages via NF-κB and PPAR-γ signaling pathways [27, 28]. Overexpression of FGF21 dramatically improved the therapeutic efficacy of MSCs in treating ischemic stroke [29]. Unlike VEGF (risk of edema) or EGF [30–32], FGF21 possesses systemic safety and endogenous compatibility, with no reported mitogenic risks in clinical trials and better endogenous feedback regulation to minimize overdose toxicity [33, 34]. Also, FGF21’s peripheral actions (e.g., hepatic lipid metabolism, cardiac protection) [35, 36] may synergize with CNS effects to improve post-stroke systemic recovery. However, the short half-life and poor stability of FGF21 limit its application [37].

Leveraging genetically attenuated virulence, we engineered a bacterial-based delivery system for FGF21 using attenuated *Salmonella typhimurium* (*S.t* -ΔpG^FGF21^). Utilizing a pBAD prokaryotic expression system, continuous FGF21 production was induced via intraperitoneal injection of L-arabinose. This approach established a targeted, sustained FGF21 delivery platform for ischemia treatment. The engineered *Salmonella* demonstrated tropism for peripheral ischemic lesions, actively multiplying at these sites. Concurrently, the neuroprotective protein FGF21 was continuously induced, enabling long-term therapy. Collectively, we developed an engineered *Salmonella* -based therapeutic strategy that exploits targeted localization, inherent non-toxicity (due to attenuation), and sustained drug release to achieve long-term treatment of ischemic stroke.

## Methods and Materials

### Plasmid construction and bacteria culture

The bacterial strain *Salmonella typhimurium* strain SHJ2037 (*S.t*-ΔpG^lux^) used in this study was kindly provided by Prof. Jin Hai Zheng from Hunan University. The recombinant expression plasmid PelB-FGF21-pBAD was constructed as previous reported [38], and electroporation method was applied for plasmid transformation [39]. The bacteria (*S.t*-ΔpG^lux^/ *S.t*-ΔpG^FGF21^) were cultured in Luria-Bertani (LB) medium aerobically at 37 ℃ with shaking at 200 rpm. The OD_600_ was measured to determine the bacterial count.

### The expression and secretion of FGF21

The overnight *S.t*-ΔpG^FGF21^ culture was diluted (1:50) into fresh LB medium at 37 ℃ with antibiotics. When *Salmonella* count arrived to an optical density (OD_600_ = 0.6), L-Arabinose (final concentration, 0.2%) was added to induce FGF21 for 6 h. The pellet strain were collected and secreted FGF21 in supernatant were precipitated through cold trichloroacetic acid method as previous reported [20].

### Cell line and cell culture

The PC-12 cell line purchased from National Collection of Authenticated Cell Cultures (SCSP-517, Shanghai, China) were cultured in Dulbecco’s Modified Eagle Medium (DMEM, Gibco, USA) supplemented with 10% fetal bovine serum (FBS, Gibco, USA) and 1% penicillin-streptomycin (Gibco, USA) in an incubator at 37 ℃, 5% CO_2_. In addition, freshly cultured bacteria were added into medium for further detection.

### Oxygen-Glucose deprivation (OGD) model

To simulate ischemic conditions in vitro, PC-12 cells were subjected to oxygen-glucose deprivation (OGD). Briefly, cultured neurons were incubated in glucose-free DMEM (Thermo Fisher Scientific) in an anaerobic chamber (95% N₂, 5% CO₂) at 37 °C for 8 h. Control cells were maintained in normal glucose-containing medium under normoxic conditions. Conditioned medium containing *S.t* -ΔpG^FGF21^-secreted FGF21 were cultured with cells for 6 h.

### Primary neuron isolation and characterization

Primary cortical neurons were isolated from new borned C57BL/6 as previously described [40]. Briefly, cortices were dissected out in ice - cold Hank’s balanced salt solution (HBSS) under a dissecting microscope. The isolated cortices were then enzymatically digested with 0.25% trypsin-EDTA, and mechanically dissociated. Neurons were plated on poly-D-lysine-coated (1.5 µg/ml) dishes in Neurobasal medium supplemented with B-27 (2%), glutamine (0.5 mM), and penicillin-streptomycin (1%). Neuronal purity was confirmed by immunocytochemistry using anti-NeuN (Ab134014, Abcam) and anti-MAP2 (78667, Cell Signaling Technology). Cultures exhibiting > 90% neuronal purity were used for experiments.

### Animals and groups

Healthy adult male C57BL/6 mice weighing 25–30 g (10–12 weeks old) were purchased from Zhejiang Vital River Laboratory Animal Technology Co., Ltd (Zhejiang, China). Mice were kept in an animal facility under a 12-hour light/dark cycle, and provided ad libitum access to water and food. The experimental protocol followed the guidelines of animal care and use of the National Institutes of Health and were approved by the Institutional Animal Care and Use Committee of Wenzhou Medical University. Before grouping and surgery operating, neurological behavioral score of 0 were recognized as completely normal. To randomly grouping, mice were weighed and recorded baseline parameters for following stratifying (25–26 g, 26–28 g, 29–30 g). With each stratum, mice were randomly assign to sham group, dMCAO group, dMCAO + FGF21 group, dMCAO + *S.t*-ΔpG^FGF21^ (-) group, dMCAO + *S.t*-ΔpG^FGF21^ (+) group (intraperitoneal injection of L-Arabinose for FGF21 induction) and dMCAO + *S.t*-ΔpG^FGF21^ +3-MA group. Randomization sequence maintained by an independent researcher. Surgeons blinded to group assignments, and outcome assessors blinded to treatment groups. FGF21 was intraperitoneally injected at a dose of 10 mg/kg. 3-MA was dissolved in PBS and injected intracranially (0.3 mg/kg, 2 µL) at 6 h post dMCAO, as previously described [41].

### Procedures for the DMCAO model

A mouse model of distal middle cerebral artery blockage was constructed by distal burning method. Briefly, mice were anesthetized by inhalation of 5% isoflurane and maintained with 2% isoflurane until the suturing was completed. After skin preparation and disinfection, the mice were placed on a thermostatic heating blanket and fixed in lateral recumbency. A 1-cm incision was made along the line between the lateral canthus of the left eye and the ipsilateral external auditory canal of the mouse, the temporal muscle was separated, and the skull was exposed. The distal branch of the middle artery of the brain was exposed after a small hole of 1 to 2 millimeters in size was made with a cranial drill. The distal middle cerebral artery was cauterized with an electrocoagulation pen, causing it to become fully occluded. A heating blanket was used to maintain the core body temperature of the mice at 36–37 ℃ during surgery until they recovered from anesthesia. *S.t*-ΔpG^FGF21^ were treated through tail vein injection after dMCAO surgery, and L-Arabinose (20%, 250 µL, for five consecutive days) was intraperitoneally given for persistent induction.

### The inclusion/exclusion criteria

Animal used in this study must undergo at least 7 days of acclimatization in the same environment, with no visible deformities or congenital diseases. Before grouping and surgery operating, neurological behavioral score of 0 were recognized as completely normal. Preoperative weight loss does not exceed 10% of baseline body weight. Animals meet the following conditions shall be excluded: (1) death within 24 h post-surgery, (2) failure to resume spontaneous eating/drinking, (3) persistent neurological deficit score of 0 (indicating model failure), (4) weight loss exceeding 25% of baseline body weight, (5) severe infection or other complications affecting results.

### Detection of plasma glucose consumption

Blood samples were collected from experimental animals and the blood glucose levels were measured using a glucometer (Yuwell, China) according to the manufacturer’s instructions.

### Optical bioluminescence imaging

Mice were intravenously injected with 100 µl of sterile saline containing 1*10^7^ CFU of *S.t*-ΔpG^lux^. After anesthetized with isoflurane (2.5% in air), bioluminescence imaging was performed with the PerkinElmer IVIS Lumina X5 (USA) at 0, 24, 72 h, 5 d, 7 d, 9 d, and 14 d after *S.t*-ΔpG^lux^ injection. The average fluorescence intensity within the region was recorded using Living Image software v4.7.2 (PerkinElmer).

### Neurobehavioral evaluations

The modified neurological severity scores (mNSS) test was performed to assess neurological deficits. The mNSS scores included sensory, motor, reflex, and balance scores. Scores ranged from 0 (normal performance) to 18 (maximum deficits), with higher scores reflecting more severe neurologic deficits. Mice with abnormal preoperative scores (> 0) were excluded. The rotarod test was used to evaluate the coordination of the mice. All mice were pretrained for 14 days prior to operation, and all researchers involved in the assessment test were unaware of the experimental grouping.

### TTC staining experiment

Intact mouse brains were isolated immediately after euthanasia 5 days and 28 days after L-Arabinose induction. The whole brains were cut into five consecutive 2 mm thick slices using a standardised rodent brain trough (RWD Life Science, China). Each slice was carefully immersed in 2.0% TTC solution (Solarbio, Beijing) and reacted at 37 ℃ for 30 min lucifugously. Infarcts were analysed by ImageJ software (National Institutes of Health, Bethesda, MD, USA). The ratio of infarct volume was calculated according to the following formula: infarct zone ratio = [(total volume of contralateral hemisphere) - (total volume of ipsilateral hemisphere staining)] / (total volume of contralateral hemisphere).

### Immunofluorescence assays

Brain sections were fixed with 4% paraformaldehyde and then incubated in PBST (0.3% TritonX-100 in PBS) containing 5% bovine serum albumin (BSA) (ST025, Beyotime, China) solution for 2 h to rupture cell membrane and inhibit nonspecific staining. Afterwards, each section was incubated with primary antibodies (PKH26 [D0030, Solarbio], FlagTag [T0053, Affinity], FGF21 [Ab171941, Abcam], NeuN [Ab104224, Abcam], Phospho-FGFR1[Ab59194, Abcam], Salmonella [Ab35156, Abcam], Iba1 [Ab5076, Abcam]), CD3 [17617-1-AP, Proteintech], and CD68 [28058-1-AP, Proteintech] at 4 ℃ overnight. To stain membrane protein, TritonX-100 was avoid to keep membrane intact. Following incubation with corresponding secondary antibodies at room temperature for 1 h, cell nuclei were labelled with DAPI (C1006, Beyotime, China) and imaged under a confocal laser scanning microscope (Leica, Stellaris 5). Five randomly selected microfields from each section were analysed. Finally, quantitative analyses were performed using Image J software.

### Western blot analysis

Proteins were extracted from brain tissue using RIPA lysis buffer (R0010, Solarbio, Beijing, China) containing PMSF protease inhibitor (P0100, Solarbio, Beijing, China). Protein products were separated by sodium dodecyl sulfate-polyacrylamide gel electrophoresis (SDS-PAGE), and then transferred onto a PVDF membrane (Millipore). After blockage with 5% skim milk for 2 h, the membranes were incubated overnight at 4 ℃ with corresponding primary antibodies (Bax [AF0120, Affinity], Bcl-2 [AF6139, Affinity, ], Cleaved-Caspase3 [Ab32042, Abcam], P62 [AF5384, Affinity], LC3B [Ab192890, Abcam], ATG5 [DF6010, Affinity], AMPKα [5832, Cell Signaling], Phospho-AMPKα [50081, Cell Signaling, ], Phospho-mTOR (Ser2448) [AF3308, Affinity], FGFR1 [AF6156, Affinity], Phospho-FGFR1 [Ab59194, Abcam]). After washing off unbound primary antibodies, the membranes were then incubated with the secondary antibody (Goat Anti-Rabbit IgG H&L (HRP) [Ab6721, Abcam) at room temperature for 1 h. Protein blots were exposed with a ChemiDoc Imaging Systerm instrument (Bio-Rad Laboratories, Inc.). The corresponding bands were analyzed using Image J software (v2.3.0).

### Tunel staining

Neural apoptosis was detected by TUNEL staining using the TUNEL Apoptosis Assay Kit (Beyotime, Shanghai, China) according to the manufacturer’s instructions. TUNEL stained the apoptotic nuclei, while 4’,6-diamino-2-phenylindole (DAPI) with fluorescein-dUTP stained all nuclei. Apoptotic cells were evaluated by observation and photography with a confocal laser scanning microscope (Leica, Stellaris 5) in five random fields in each section. The apoptotic index was calculated as the percentage of TUNEL-positive cells among number of the NeuN stained cells using ImageJ software.

### Enzyme-linked immunosorbent assay

Serum levels of IL-6, IL-1β, TNF-α, MCP-1, IFN-γ and FGF21 were measured using an enzyme-linked immunosorbent assay (ELISA) kit (Boyun Biotechnology, China). Blood was extracted from the abdominal aorta in deeply anesthetized mice and placed at room temperature for 0.5 h. After condensation, blood samples were centrifuged at 4℃ 2000 rpm for 20 min to obtain serum. Optical density (OD) was measured at wavelength of 450 nm by using SpectraMax 190 Microplate Reader (Molecular Devices, San Jose, CA, USA ).

### Quantitative Real-Time PCR (qRT-PCR)

Total RNA was extracted using TRIzol reagent (Invitrogen) and reverse-transcribed into cDNA with a High-Capacity cDNA Reverse Transcription Kit (Takara). qRT-PCR was performed using SYBR Green Master Mix (Takara) on a QuantStudio 3 Flex system (Thermo Fisher Scientific). Gene expression was normalized to GAPDH and calculated via the 2^(-ΔΔCt) method. Primer sequences: *Glut1*: Fwd 5’- TCGTCGTTGGCATCCTTATT’, Rev 5′-TGCAGGTCTCGGGTCACAT-3′.

### Small interfering RNA transfection

For FGFR1 silencing, small interfering RNA (siRNA)-FGFR1, and riboFECT CP reagents (C10511-1, RiboBio Co., Ltd., China) were used for the transfection of PC12. Firstly, PC12 cells were incubated in 6-well plates with 1*10^6^ cells/well and followed by blending siRNA-FGFR1 (5 µl; three targeted sequences: siRNA-FGFR1-001, CACCTACTTCTCCGTCAATGTCT; siRNA-FGFR1-002, GAGCATCATAATGGATTCTGTGG; siRNA-FGFR1-003, TCGAGACATTCATCATATCGACT) into 1×riboFECT CP buffer and ribo-FECT CP reagent to form a transfection mixture. PC12 cells were then transfected with the mixture and cultured in DMEM for 72 h. NC siRNA (targeted sequence: GGCTCTAGAAAAGCCTATGC) was used for the negative control. Based on western blot analysis, the most effective sequence of siRNA-FGFR1 was selected for subsequent experiments.

### Hepatic and renal function

Blood samples were collected from all animal groups after intravenous injection of bacteria. Biochemical analyses were performed on blood specimens after centrifugation to assess hepatic and renal toxicity after bacterial injection. Blood levels of aspartate aminotransferase (AST) and alanine aminotransferase (ALT) indicators reflecting liver function, and levels of urea nitrogen (BUN) and creatinine (CREA) indicators reflecting kidney function were measured. Body weight of the mice was tested for every three days after intravenous injection of *Salmonella*.

### Hematoxylin and Eosin (H&E) staining

The dehydrated and cleared tissues were embedded in paraffin blocks and sectioned into 4–6 μm thick slices using a microtome. Sections were placed on glass slides and dried at 60 ℃ for 1–2 h. After that, sections were deparaffinized in xylene and rehydrated through a graded series of ethanol (100%, 95%, 90%, 80%, and 70% ethanol) to distilled water. The sections were stained with hematoxylin for 5–10 min, rinsed with tap water, and then differentiated in 1% acid alcohol (1% HCl in 70% ethanol) for a few seconds. After rinsing with tap water again, the sections were blued for 2–3 min. The sections were then stained with eosin for 1–2 min, dehydrated through a graded series of ethanol (95%, 100% ethanol), cleared in xylene, and mounted with a coverslip using a mounting medium. Morphological changes were analyzed under a light microscope (Olympus BX53F).

### Statistical analysis

All statistical analyses for this study were performed using GraphPad Prism 9. Data were expressed as mean ± standard deviation (SD). Differences between the two groups were assessed using an unpaired two-tailed Student’s *t*-test. Comparisons between multiple groups were performed using a one-way analysis of variance (ANOVA). *p*-value < 0.05 was considered as statistically significant.

## Results

1. ***Salmonella*targets and colonizes cerebral infarcted area**.

**Fig. 1:**
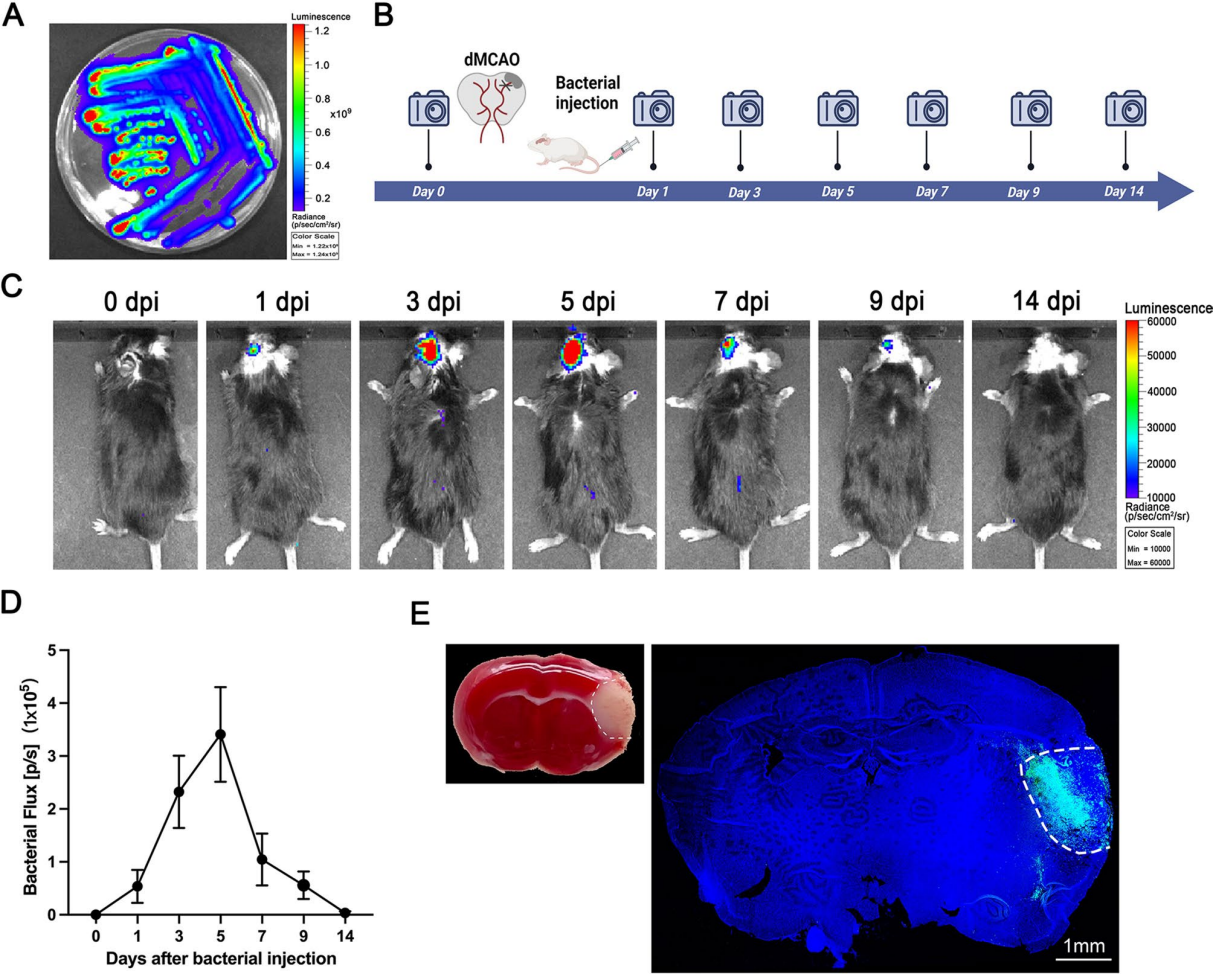
*Salmonella* (*S.t*-ΔpG^lux^) Targets to cerebral infarct zones. **(A)** Bioluminescent signal of *Salmonella* (*S.t*-ΔpG^lux^) on LB plates; (**B**, **C**, **D**) Experimental timeline and bioluminescence images of animals after injection with *Salmonella* (*S.t*-ΔpG^lux^). Images were captured and quantified on 1 d, 3 d, 5 d, 7 d, 9 d, and 14 d after injection, *n* = 6; (**E**) Global scanning of luminous *Salmonella* captured from the ischemic semi-dark band. *Salmonella* was labelled in green, and nuclei were stained in blue, scale bar = 1 mm, *n* = 6

Given the attenuated *Salmonella typhimurium*’s (*S.t* -ΔpG) confirmed ability to cross the blood-brain barrier (BBB) and proliferate in orthotopic gliomas [20], we hypothesized its facultative anaerobic metabolism would promote preferential colonization of hypoxic/necrotic regions. To noninvasively track bacterial distribution in vivo, the *lux* operon was integrated into the chromosome of engineered *Salmonella* (*S.t*-ΔpG^lux^) to produced bioluminescence automatically. The luminescence signal on LB plates confirmed that *S.t*-ΔpG^lux^ gains the ability to glow autonomously (Fig. 1A). Then the individual colonies was cultured and intravenously injected into mice with established dMCAO model of stroke. After examined the bioluminescence by an in vivo imaging system, the result showed the engineered *Salmonella* accumulation within infarcts. Bioluminescence progressively increased, peaking at 5 days post-injection (dpi) before declining by 7 dpi and resolving within ~ 2 weeks (Fig. 1B-D), indicating sustained proliferation within ischemic tissue.

The specificity of the bacteria for infarct foci was verified by immunofluorescence staining after brain sampling in mice sacrificed at 5 dpi. The result showed that dense bacterial clusters localized exclusively to infarcted regions, with no colonization in healthy tissue (Fig. 1E). Based on this phenomenon, we explored the possibility of *Salmonella* as a vector for targeted therapeutic delivery to cerebral ischemic areas.

2. **Engineering FGF21-producing*Salmonella***.

**Fig. 2:**
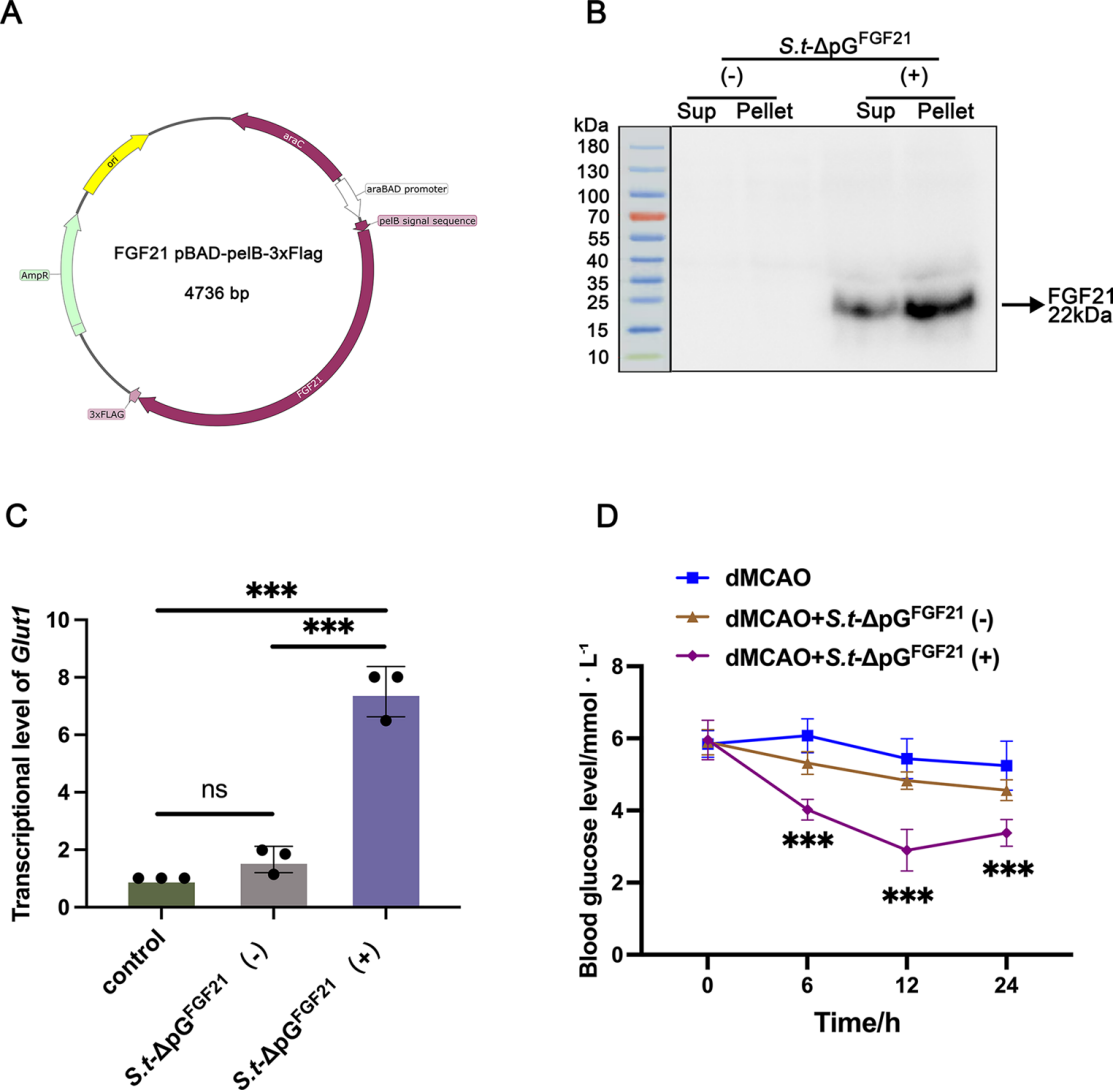
Engineering and induction of FGF21-expressing bacteria (*S.t*-ΔpG^FGF21^) in vitro. (**A**) Genomic map of the engineered plasmid pBAD-PelB-FGF21-3*Flag; (**B**) Immunoblot analysis of bacterially secreted FGF21. Samples were prepared with (+) or without (-) 0.2% L-Arabinose and separated into pellet and supernatant (Sup) fractions. (**C**) Quantitative transcription analysis of *gult1* upon bacterial secreted FGF21 in PC-12 cells, *n* = 3. (**D**) Global glucose level determined on 0, 6, 12, and 24 h after bacterial injection, *n* = 5. ** *p* < 0.01, and *** *p* < 0.001; ns, no significance

To generate an inducibly secretable vector system for bacterial expression of therapeutic genes, the FGF21 fraction was fused to the PelB leader sequence into a pBAD vector (PelB-FGF21-3*flag-pBAD), confirmed by sequencing. After that, the successful PelB-FGF21-3*flag-pBAD vecter was transferred into the attenuated *Salmonella* through electrotransformation (Fig. 2A). The inducer L-Arabinose with final concentration of 0.2% was used to produce target protein. Western blotting analysis revealed that FGF21 protein (22 kDa) was abundantly expressed in the sediment and medium supernatant of the strain carrying pFGF21 after L-Arabinose induction compared with no induction group (Fig. 2B). This result suggested that FGF21 expression was specifically induced and could be secreted and released from bacteria benefitted by the pelB transmembrane peptide.

To validate bioactivity, we assessed FGF21’s metabolic function (a known enhancer of glucose uptake). In vitro, L-Arabinose-induced bacterial FGF21 significantly enhanced *glut1* transcription versus non-induced controls (Fig. 2C), confirming enzymatic competence. In vivo, bood glucose kinetics showed that *S.t*-ΔpG^FGF21^ (+) administration reduced blood glucose by 33.75% at 6 h post-induction, significantly exceeding the 32.05% reduction in non-induced controls (*p* < 0.001; Fig. 2D). These data confirmed that the FGF21 protein secreted by *Salmonella* retains biological enzymatic activity.

3. **Engineered*Salmonella*improves neurological deficits and reduces infarct volume after dMCAO in stroke mice**.

**Fig. 3:**
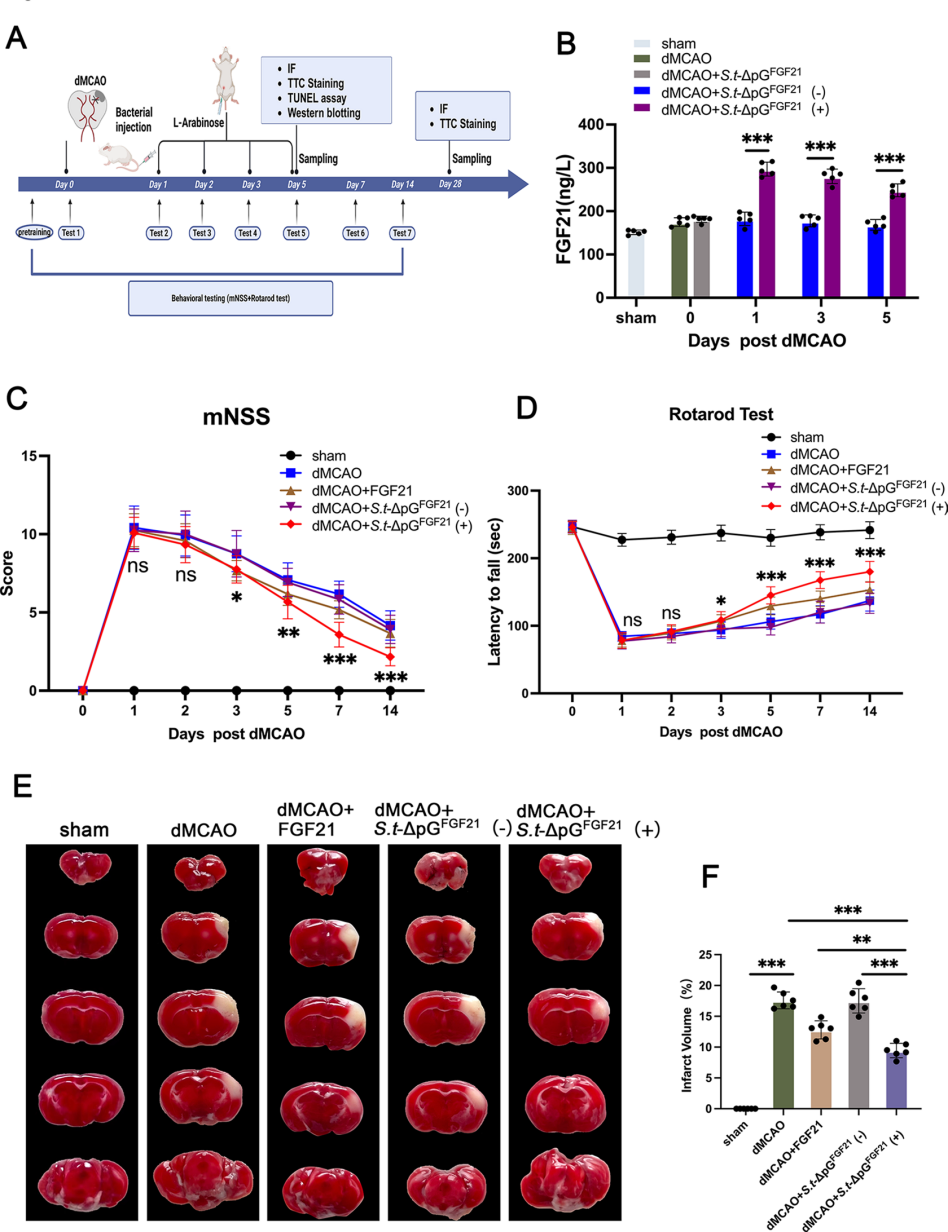
*S.t*-ΔpG^FGF21^(+) ameliorates neurological deficits and reduces the volume of cerebral infarct foci in stroke mice. (**A**) Experiments timeline; (**B**) ELISA analysis of FGF21 expression in vivo, *n* = 5. (**C**) mNSS, *n* = 12; (**D**) Rotarod test, *n* = 12; (**E**) Representative images of TTC stained brain slices on 5 days after dMCAO; (**F**) Quantitative analysis of brain infarct volumes, *n* = 6. * *p* < 0.05, ** *p* < 0.01, and *** *p* < 0.001; ns, no significance

Previous reports have confirmed recombinated hFGF21 could alleviate neuroinflammation and improves neurological outcomes following focal ischemic stroke [25–27]. To evaluate whether bacterially secreted FGF21 could improve neurological deficits in dMCAO model, the attenuated *Salmonella* (*S.t*-ΔpG^FGF21^ ) was intravenously injected 6 h after stroke induction. The L-Arabinose administration was given 24 h post bacterial injection and maintained for 5 consecutive days thereafter (Fig. 3A). Serum FGF21 levels were longitudinally monitored at postoperative days 1, 3, and 5 post stroke.As shown in Fig. 3B, the MCAO group showed mild FGF21 elevation versus sham, likely reflacting endogenous stress responses, but returned to baseline by day 5. The dMCAO + *S.t*-ΔpG^FGF21^ (+) exhibited rapid FGF21 surge (1.7-fold higher than model group at day 1, *p* < 0.01), sustaining > 1.4-fold elevation through day 5 (*p* < 0.01). *S.t*-ΔpG^FGF21^ (-) group mirrored the dMCAO group, confirming strict induction-dependency. Neurological deficits and feeling function were assessed by the modified neurological severity score (mNSS) test and Rotarod test. As shown in Fig. 3C, the dMCAO + *S.t*-ΔpG^FGF21^ (+) group exhibited better neurological recovery since day 3 post *Salmonella* injection. On day 14 post stroke, the mNSS score of dMCAO + *S.t*-ΔpG^FGF21^ (+) group was significantly better than those in the dMCAO group and *S.t*-ΔpG^FGF21^ (-) group; Similarly, the dMCAO + *S.t*-ΔpG^FGF21^ (+) group exhibited a significant improvement in enhanced motor coordination, indicated by an increased latency time to fall off the rotarod, comparing to the non-induced group at 14 days after dMCAO (Fig. 3D). These data indicated the neurological recovery with induced *S.t*-ΔpG^FGF21^ administration. Critically, *S.t*-ΔpG^FGF21^ (+) significantly reduced infarct volume at day 5 (9.45 ± 1.17% vs. 17.58 ± 1.36% in dMCAO and 17.50 ± 1.98% in non-induced controls; *p* < 0.01; Fig. 3E, F). Although the FGF21 treatment group also exhibited certain neuroprotective effects, it was not comparable with *S.t*-ΔpG^FGF21^ (+) group. These results demonstrated that FGF21 secretion-not bacterial colonization alone mediated neuroprotection.

4. **Engineered*Salmonella*prevents neuronal apoptosis in stroke mice**.

**Fig. 4:**
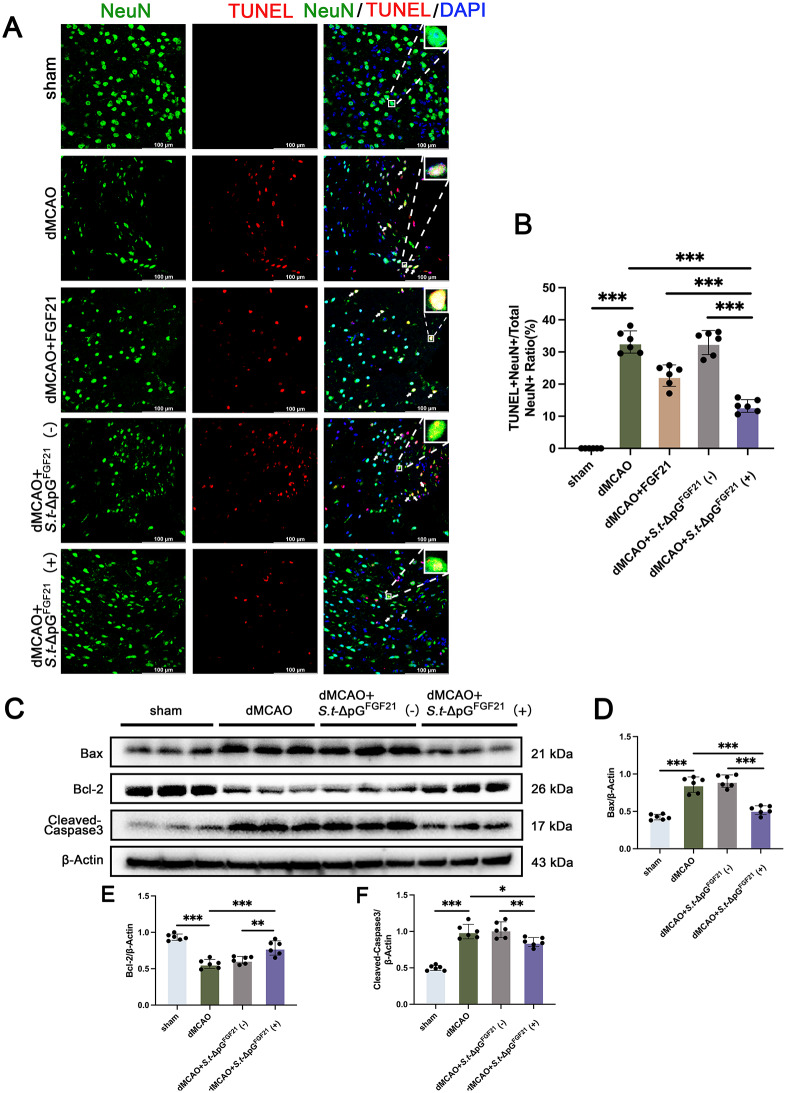
*S.t*-ΔpG^FGF21^(+) improves neuronal apoptosis after stroke. (**A**) Representative images of TUNEL and NeuN co-staining in different groups, scale bar = 100 μm; (**B**) Quantitative analyses of neuronal apoptosis rate in each group, *n* = 6; (**C**-**F**) Expression level and relative quantitative analysis of Bax, Bcl-2, and Cleaved-Caspase3, *n* = 6. * *p* < 0.05, ** *p* < 0.01, *** *p* < 0.001

Subsequently, NeuN and TUNEL double staining was performed to evaluate cortical neuronal apoptosis around infract aera 5 days post IS. Compared with the sham group, the ischemic injury resulted in a significant neuronal apoptosis, approximately 33.12 ± 3.47%, which was not significantly different from the percentage in the *S.t*-ΔpG^FGF21^ (-) group ( 32.93 ± 3.75%), whereas *S.t*-ΔpG^FGF21^ (+) increased the neuronal survival to 13.22 ± 1.98% after L-Arabinase administration (Fig. 4A-B), confirming effective neuronal anti-apoptotic activity of *S.t*-ΔpG^FGF21^. To further confirm the anti-apoptotic effect, we performed western blotting to examine apoptotic proteins. As expected, induced *S.t*-ΔpG^FGF21^ (+) administration could availably inhibit mature of Caspase-3 and reduce Bax expression, along with stable expression of Bcl-2 (Fig. 4C-F). Notably, bacterially secreted FGF21 outperformed signle dose FGF21 administration (15 mg/kg, ip) in reducing apoptosis (Figure S1), likely due to sustained local delivery. These data established that FGF21-secreting *Salmonella* inhibited apoptosis in ischemic brain tissue.

5. **Engineered*Salmonella*improves long-term recovery after ischemic stroke**.

**Fig. 5:**
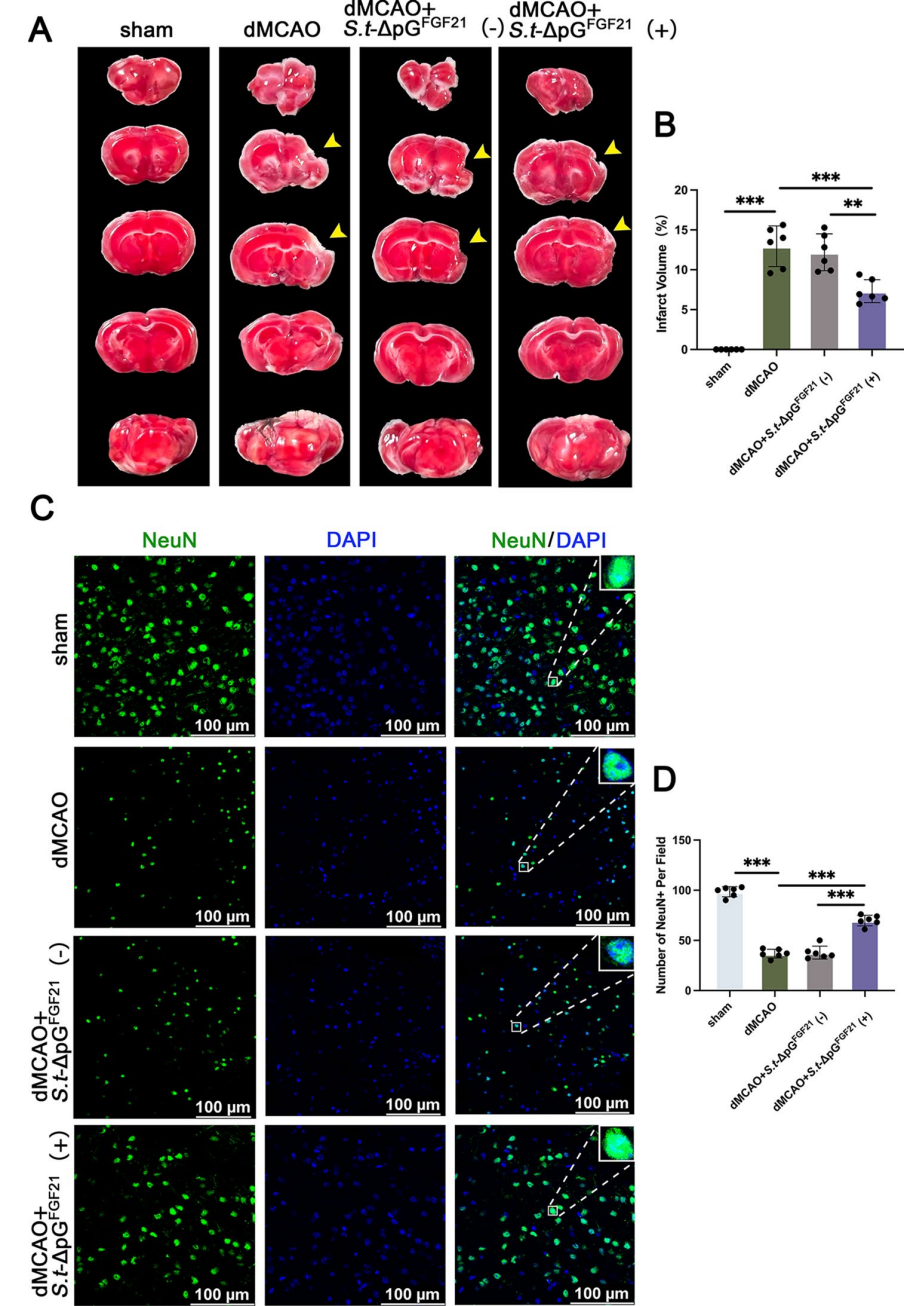
*S.t*-ΔpG^FGF21^(+) promotes long-term stroke recovery in mice model. (**A**) Representative images of TTC stained brain slices on 28 days after dMCAO; (**B**) Quantitative analysis of brain infarct volumes, *n* = 6; (**C**) Representative images of NeuN staining in different groups, scale bar = 100 μm; (**D**) Quantitative calculation of surviving neurons in each group, *n* = 6. ** *p* < 0.01, *** *p* < 0.001

Long-term stroke recovery was examed by TTC staining at day 28. The results showed significant cerebral cortical atrophy in the chronic phase after stroke compared to cortical oedema in the acute phase. No significant difference was observed between dMCAO group (12.96 ± 2.54%) and dMCAO + *S.t*-ΔpG^FGF21^ (-) group (12.20 ± 2.31%), suggesting that bacterial grafting alone does not contribute to long-term neurological improvement after stroke. In contrast, the dMCAO + *S.t*-ΔpG^FGF21^ (+) group showed a significant reduction in the area of cerebral cortical atrophy, which was approximately 7.32 ± 1.45% (Fig. 5A-B). Therefore, bacterial-induced secretion of FGF21 is effective in maintaining the integrity of the cerebral cortex and reducing cortical atrophy in the chronic phase after ischemic stroke, with long-term effectiveness. We subsequently performed NeuN staining to assess the survival of cortical neurons around the infarcted foci 28 days after stroke. Ischemic injury resulted in a significant reduction in the number of neurons compared with the sham-operated group, with no significant difference compared with the *S.t*-ΔpG^FGF21^(-) group, whereas bacterial treatment with *S.t*-ΔpG^FGF21^(+) significantly increased the number of NeuN-positive cells (Fig. 5C-D). These data confirm that bacterial-induced secretion of FGF21 is effective in increasing cortical neuronal survival after ischemia in the long-term stroke recovery.

### Bacterial exclusion from neuronal cells

**Fig. 6:**
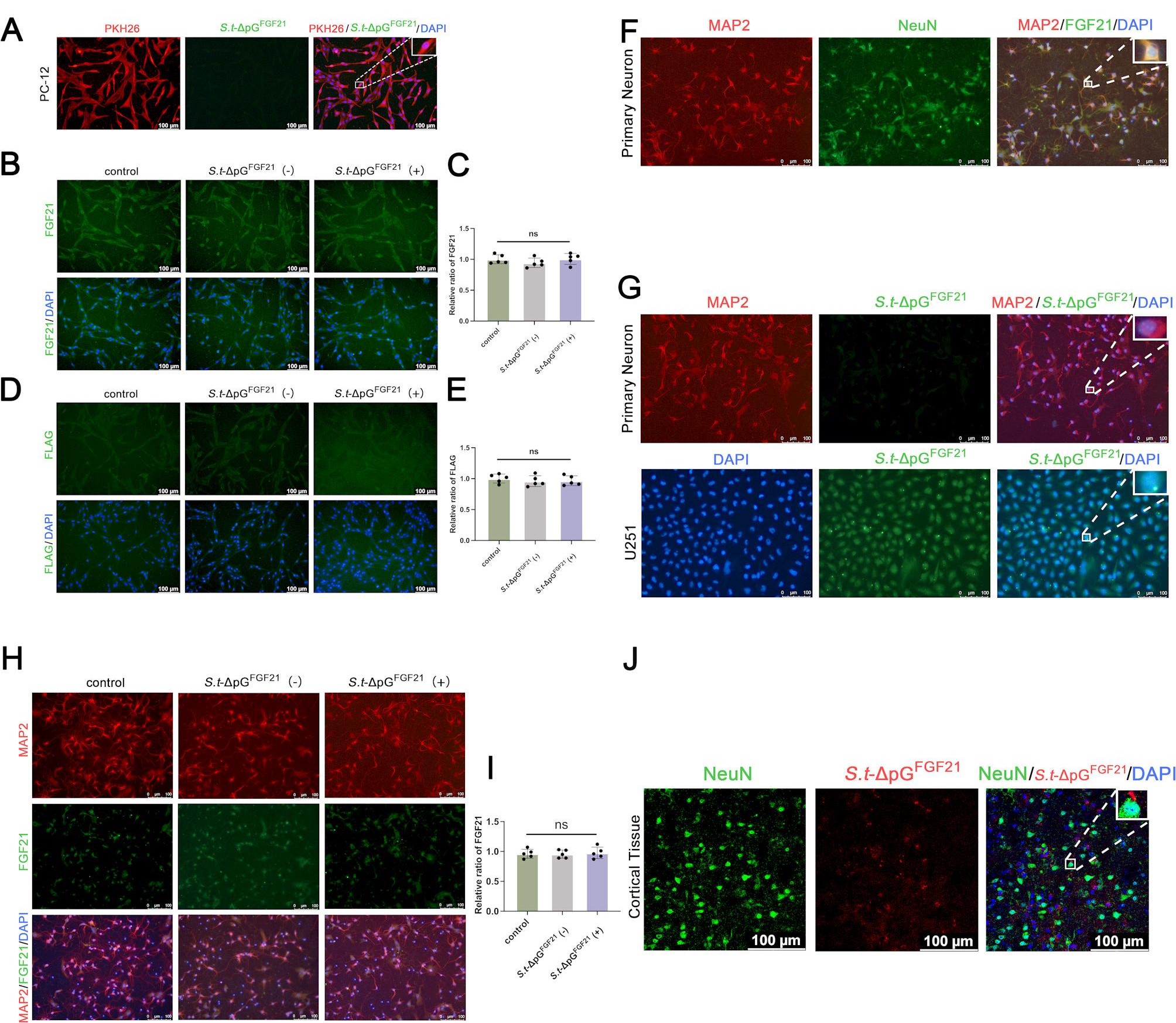
*Salmonella* was excluded from Neuronal Cells. (A) Co-culture of PC-12 cells and *Salmonella* (*S.t*-ΔpG ^FGF21^). PKH26 (red) labelling of cell membranes and anti-*Salmonella typhimurium* LPS antibody labelling *Salmonella* (green), *n*=5, scale bar = 100 μm; (B-C) Immunofluorescence staining and quantitative analyses of FGF21 protein (green), *n*=5, scale bar = 100 μm; (D-E) Immunofluorescence staining and quantitative analyses of Flag fragment (green), *n*=5, scale bar = 100 μm; (F) Identification of primary neuron with MAP2 (red) and NeuN (green). Positive cells are greater than 90%; (G) Co-culture of primary neuron cells and U251 cells with *Salmonella* (*S.t*-ΔpG ^FGF21^). MAP2 (red) labelling of neuronal cytoplasm and anti-*Salmonella typhimurium* LPS antibody labelling *Salmonella* (green), *n*=5, scale bar = 100 μm; (H-I) Immunofluorescence staining and quantitative analyses of FGF21 protein (green) in primary neuron cells, *n*=5, scale bar = 100 μm; (J) Immunofluorescence analysis of co-stained neurons (NeuN) with *Salmonella* in cortex. *n*=5, scale bar = 100 μm.

FGF21 activates multiple FGFRs, and binds FGFR1 with a much higher affinity than the other FGFR subtypes in the presence of β-klotho [42, 43]. To investigate the precise mechanism of *Salmonella* secreted FGF21 in the intracranial, we firstly culture PC-12 cells with *S.t*-ΔpG^FGF21^ to seek whether *Salmonella* could enter into cell in vitro. *Salmonella* were stained with specific antibody and PKH26 was used to label cell membrane. As shown in Fig. 6A, no obvious stained *Salmonella* existed intracellular. Intracellular FGF21 levels also showed no significant increase post L-Arabinose induction versus controls (Fig. 6B-C). Furthermore, Flag-tagged FGF21 exhibited no intracellular fluorescence (Fig. 6D-E). These data indicate *Salmonella* and its secreted FGF21 do not enter neural cells. Primary cortical neurons (P0-P1 mice; >90% MAP2^+^ /NeuN^+^ purity) similarly showed no *S.t* -ΔpG^FGF21^ internalization after 6 h co-culture (Fig. 6F-I). Conversely, the bacteria efficiently invaded U251 glioma cells under identical conditions.

To further explore whether *Salmonella* can enter neural cells in vivo, we co-stained neurons (labeled by NeuN) with *Salmonella*. The results clearly demonstrated that *Salmonella* could not enter neuronal cells in vivo (Fig. 6J). However, we observed co-localization of *Salmonella* with GFAP-positive astrocytes and IBA1-positive microglia (data not shown). These results confirmed that *Salmonella* could not enter neuron in cerebral cortex.

### Bacterially secreted FGF21 exerts anti-apoptotic effects by activating the neuronal FGFR1/AMPK/mTOR pathway to enhance autophagy

**Fig. 7:**
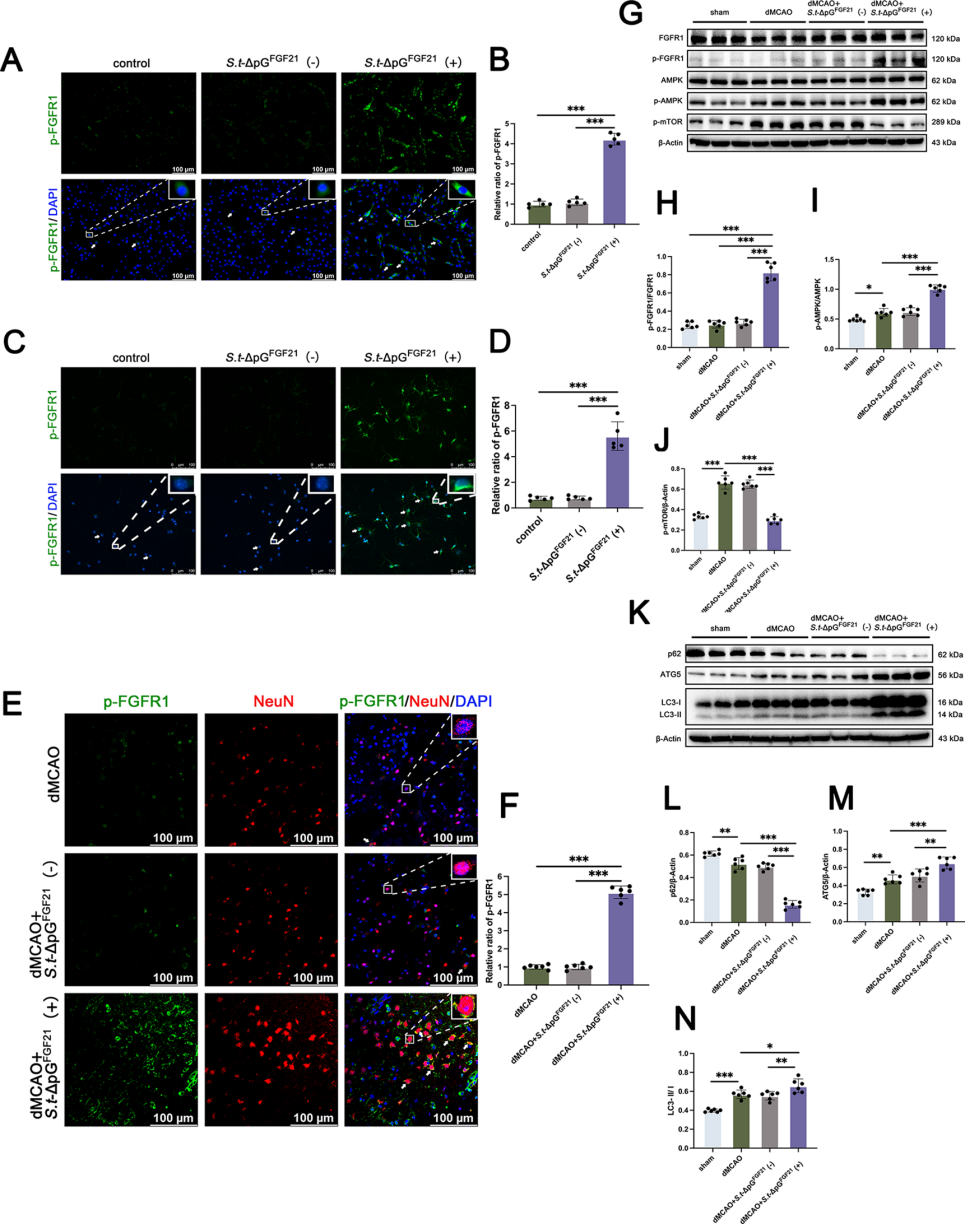
*S.t*-ΔpG^FGF21^(+) enhances autophagy through binding to the FGFR1 receptor to activate the AMPK-mTOR pathway. (**A**-**B**) Immunofluorescence staining and quantitative analyses of p-FGFR1 (green) in PC-12 cells, *n* = 5, scale bar = 100 μm; (**C**-**D**) Immunofluorescence staining and quantitative analyses of p-FGFR1 (green) in primary neuron cells, *n* = 5, scale bar = 100 μm; (**E**-**F**) Representative images of p-FGFR1 (green) and NeuN (red) staining on day 5 in the cerebral cortex, *n* = 6, scale bar = 100 μm. (**G**-**J**) Expression level and relative quantitative analysis of FGFR1, p-FGFR1, AMPK, p-AMPK, and p-mTOR in the cerebral cortex on day 5 post stroke, *n* = 6; (**K**-**N**) Representative western blot images and relative quantification analysis of autophagy relative protein including p62, ATG5, and LC 3 in the cerebral cortex of each group, *n* = 6. * *p* < 0.05, ** *p* < 0.01, *** *p* < 0.001; ns: no significant difference

As the former result, we speculated secreted FGF21 might act via membrane receptors. To verify this conjecture, we firstly co-cultureed the *Salmonella* with PC-12 and primary neuron cells along with 6 h continuous induction. The immunofluorescence staining showed significantly higher expression level of p-FGFR1, by comparing with the control group and the non-induced group (Fig. 7A-D).

To confirm the activation role of bacterially secreted FGF21 on FGFR1 in vivo, we performed immunofluorescence staining and protein blotting analysis on mice sacrificed at 5 day post dMCAO. Immunofluorescence results in the infarcted area showed that the p-FGFR1 expression level was significantly higher after L-Arabinose induction than in the dMCAO group and the non-induced group (Fig. 7E-F), which was consistent with the results of in vitro test.

As rFGF21 modulates mitophagy via AMPK [44, 45], we hypothesized that becteria secreted FGF21 may regulate the activation of AMPK/mTOR pathway to inhibit neuron apoptosis. To further investigate the relevant mechanisms after FGFR1 receptor activation, we detected the AMPK/mTOR pathway, which is closely related to cellular autophagy. The results showed that the ratio of p-AMPK/AMPK was significantly elevated after dMCAO injury, whereas the activation of mTOR was inhibited (Fig. 7G-J), which may be related to the activation of autophagy to maintain intracellular homeostasis by degrading damaged proteins and recycling cellular components under stress after cell injury. In contrast, the elevated ratio of p-AMPK/AMPK and the reduced level of p-mTOR after bacterial induction were more significant. This result confirmed the activation of the AMPK/mTOR pathway and the presence of autophagy induction. We then observed a significant increase in the expression of ATG5 and an elevated LC3-II/LC3-I ratio in the bacterially induced group, whereas the expression of p62 was significantly reduced (Fig. 7K-N). Thus bacteria enhanced cellular autophagy by secreting FGF21 acting on the FGFR1 receptor in neuronal cells and activating the AMPK/mTOR pathway.

### FGFR1 signaling is essential for FGF21-Mediated autophagy and Anti-Apoptosis

To verify the essential role of FGFR1 in FGF21-mediated pathways, PC-12 cells were pretreated with FGFR1 siRNA or scrambled control, followed by OGD and incubation with non-induced *Salmonella* lysate or L-arabinose-induced *Salmonella* lysate (FGF21+). FGF21 + increased LC3B-II/I (2.07-fold, *p* < 0.01) and ATG5 (2.58-fold, *p* < 0.01), while reducing P62 (43%, *p* < 0.05). FGFR1 silencing reverted autophagy markers to OGD baseline (Fig. S2). By detecting apoptosis suppression, FGF21(+) decreased Bax (32%, *p* < 0.01) and P17 (42%, *p* < 0.01), while elevating Bcl-2 (1.75-fold; *p* < 0.001). This demonstrated FGFR1-dependent activation of autophagy and suppression of apoptosis.

### Autophagy Inhibition reverses the role of FGF21 in post-stroke neuroprotection

**Fig. 8:**
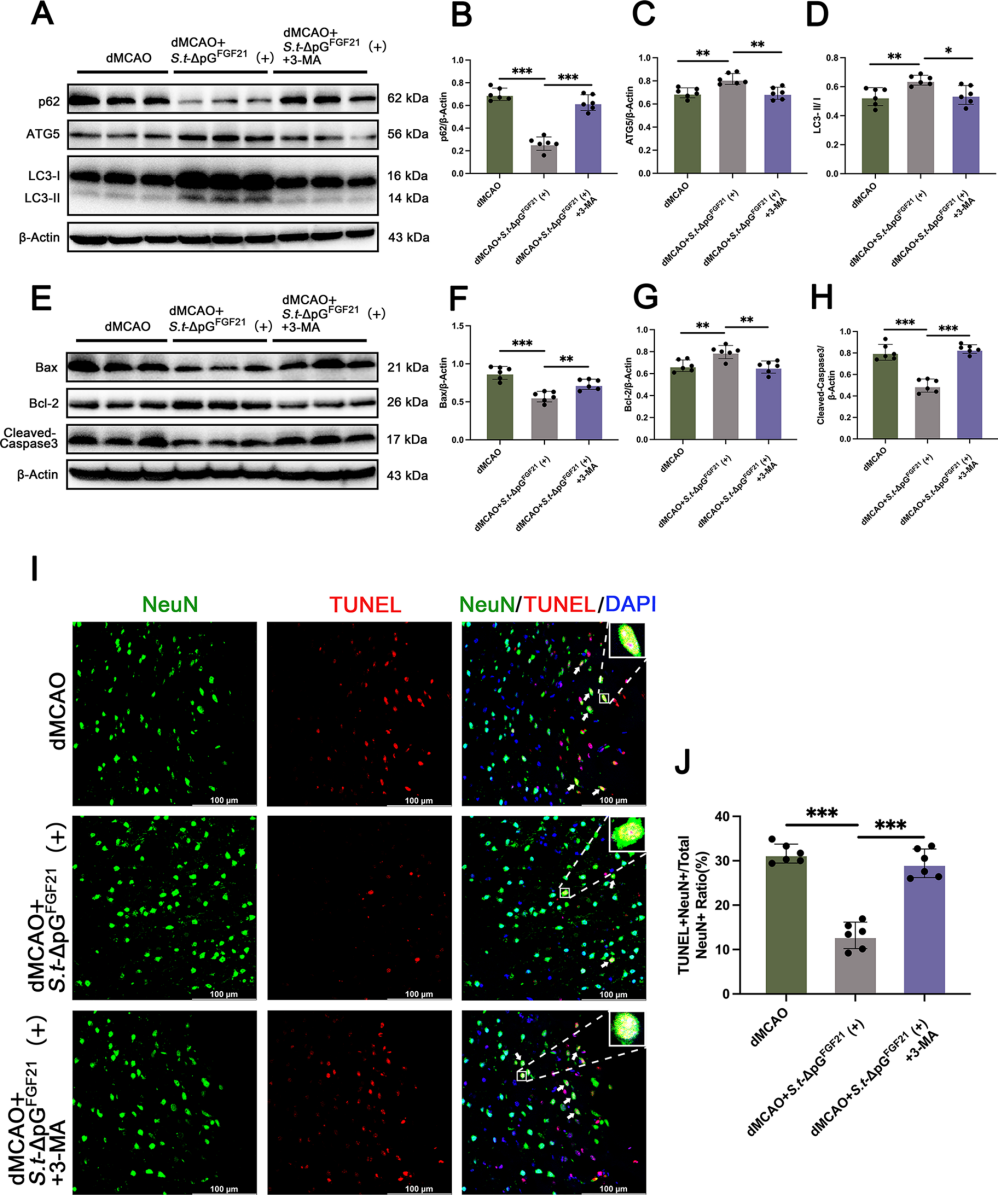
Autophagy inhibition reverses the effect of bacterially secreted FGF21 on neuroprotection after stroke. (**A**-**D**) Expression level and relative quantification of p62, ATG5, and LC3 expression in the cerebral cortex of different groups, *n* = 6. (**E**-**H**) Expression level and relative quantification of Bax, Bcl-2, and Cleaved-Caspase3, *n* = 6; (**I**-**J**) Representative images and quantitative analysis of apoptosis rate of neuronal cells in each group, scale bar = 100 μm, *n* = 6. * *p* < 0.05, ** *p* < 0.01, *** *p* < 0.001

To determine if autophagy mediates FGF21’s anti-apoptotic effects, we inhibited autophagy with 3-MA. Pre-treatment confirmed 3-MA effectively suppressed ATG5 expression and LC3-II/LC3-I ratio, along with increased p62 level (Figure S3A-D). The immunoblotting results also revealed exacerbated apoptosis marked by increased apoptitic protein, including Bax and cleaved-caspase-3 (p17), along with reduced level of Bcl-2, which suggested that autophagy occurring under stress after dMCAO could be blocked by 3-MA and led to further aggravation of cell injury (Figure S3E-H). Then we further analyzed the effect of autophagy on the therapeutic effect of FGF21. The results of protein immunoblotting showed a significant decrease in ATG5 expression and LC3-II/LC3-I ratio, along with a significant increase in p62 expression in the dMCAO + *S.t*-ΔpG^FGF21^(+) + 3-MA group (Fig. 8A-D). Compared with the dMCAO + *S.t*-ΔpG^FGF21^(+) group, the dMCAO + *S.t*-ΔpG^FGF21^(+) + 3-MA group showed a significant increase in the expression of Bax and p17, and a significant decrease in the expression of Bcl-2 (Fig. 8E-H). To further elucidate the effect of autophagy on neuronal apoptosis, dual-fluorescence of TUNEL and NeuN were used to evaluate neuron apoptosis. The result showed addition of 3-MA reverse the anti-apoptotic effect of *S.t*-ΔpG^FGF21^ administration (Fig. 8I-J). These results indicated that 3-MA significantly reversed the anti-apoptotic effect of FGF21, suggesting that bacterially secreted FGF21 promoted autophagy to exert its anti-apoptotic effect against dMCAO-induced injury.

### Assessment of immune cells infiltration in the infarcted area and systemic inflammatory indices

**Fig. 9:**
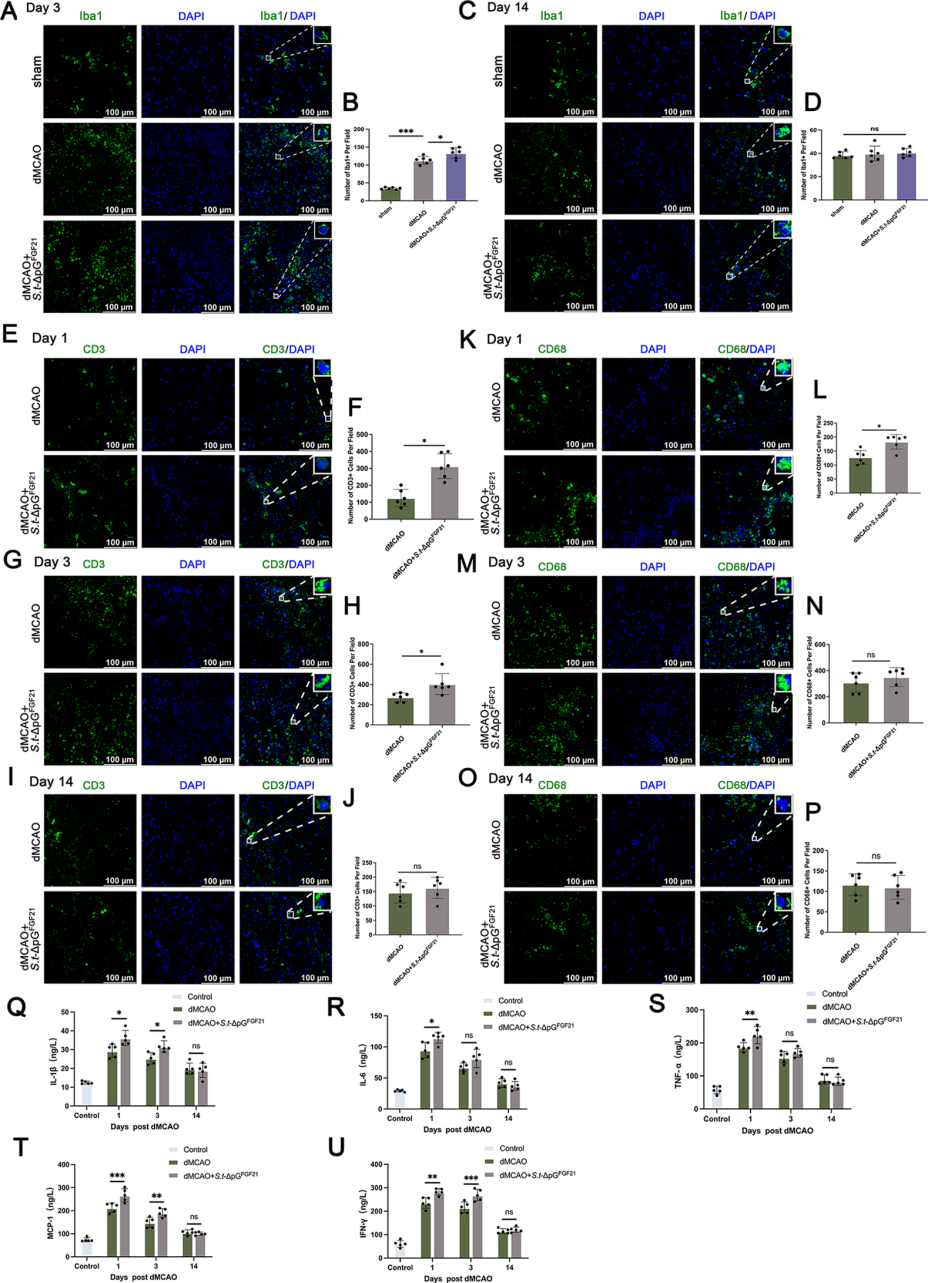
Assessment of Immune cells infiltration around cerebral infarction and detection of systemic levels of inflammatory cytokines in mice. (**A**-**B**) Representative images and quantitative analysis of Iba1 staining on 3 days after dMCAO, *n* = 6, scale bar = 100 μm; (**C**-**D**) Representative images and quantitative analysis of Iba1 staining on 14 days after dMCAO, scale bar = 100 μm; (**E**-**J**) Representative images and quantitative analysis of CD3 staining on 1, 3, 14 days after dMCAO, *n* = 6, scale bar = 100 μm; (**K**-**P**) Representative images and quantitative analysis of CD68 staining on 14 days after dMCAO, *n* = 6, scale bar = 100 μm; (**Q**-**U**) Quantification of IL-1β, IL-6, TNF-α,MCP-1 and IFN-γ in each group was assessed by ELISA, *n* = 5. * *p* < 0.05, ** *p* < 0.01, *** *p* < 0.001; ns: no significant difference

Microglial activation and phagocytosis critically regulate post-stroke brain homeostasis. Immunofluorescence revealed significantly elevated Iba1^+^ cell density in both dMCAO and bacterial-treated groups versus sham controls at day 3 post-stroke (*p* < 0.05), with further amplification in *S.t* -ΔpG^FGF21^ (+) treated mice (*p* < 0.05; Fig. 9A-B). By day 14, Iba1^+^ cell counts decreased to sham levels across all groups (Fig. 9C-D), indicating transient microglial activation without chronic CNS perturbation.

To evaluate potential immunogenicity induced by bacterial components, we performed immunofluorescence staining with CD3 and CD68 to observe the distribution of intracranial macrophages and T-cell infiltration after dMCAO and bacterial injection [46]. Data revealed the infiltration of macrophages and T-cells corroborated enhanced peripheral immune cell infiltration at 24 h post bacterial injection, resolving to model-comparable levels by day 14 (Fig. 9E-O). This result indicated that bacterial injection can exacerbate the infiltration of peripheral immune cells to some extent, but no significant difference was observed in the later stage.

Systemic inflammation was assessed via serum cytokines. pronounced elevations in IL-1β, IL-6, TNF-α, MCP-1, and IFN-γ at day 1 post-stroke, exacerbated by bacterial injection (Fig. 9Q-U). By day 14, cytokine levels normalized to baseline.

These results suggestted bacterial injection exacerbate the inflammatory response in the acute phase. However, this elevation was transient and did not cause long-term inflammatory response to the organism.

## Toxicity of bacterial infection

**Fig. 10:**
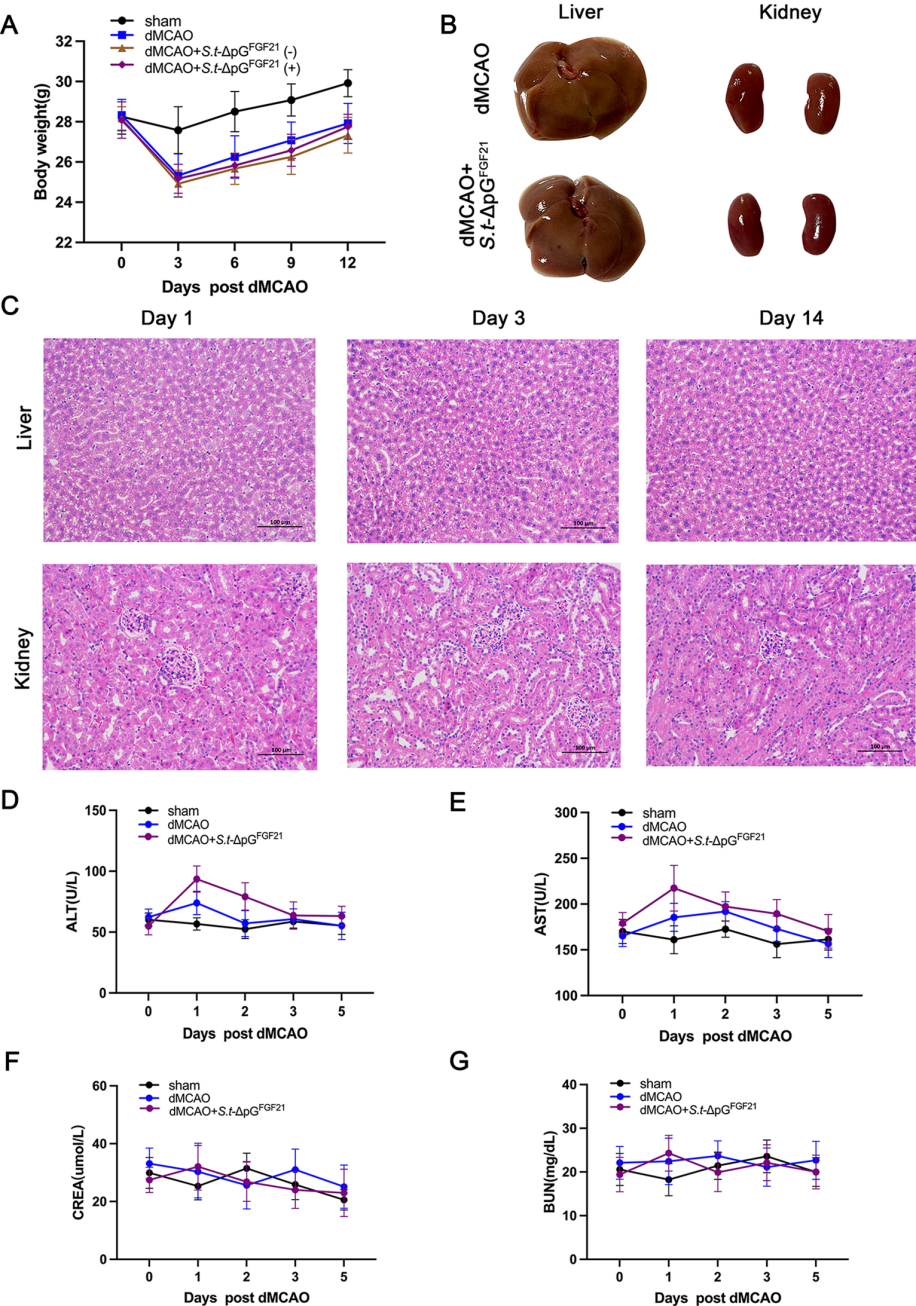
Effect of *S.t*-ΔpG^FGF21^ infection on body weight and quantification of liver and kidney functions. (**A**) Body weights of different groups of mice on 3, 6, 9, and 12 days after bacterial injection, *n* = 12; (**B**) Fresh morphology of liver and kidney tissue, *n* = 6; (**C**) Histopathological analysis of liver and kidney tissue on 1, 3, 14 days after bacterial injection, *n* = 6, scale bar = 100 μm; (**D**-**E**) Liver function determined on 1,2,3,5 days after bacterial injection, *n* = 9. The normal reference values of ALT were 10.06–96.47 U/L, and the normal reference values of AST were 36.31-235.48 U/L; (**F**-**G**) Kidney function of different groups of mice determined on 1,2,3,5 days after bacterial injection, *n* = 9. Normal reference range of CREA: 10.91–85.09 umol/L; Normal reference range of BUN: 10.81–34.74 mg/dL.

To evaluate the effect of *Salmonella* infection on the mouse organism, physiological indices, including body weight, were monitored. Body weight recovery kinetics mirrored dMCAO-only controls, stabilizing by day 12 (Fig. 10A). Histopathological analysis of liver and kidney tissue via H&E staining revealed no structural lesions or cellular abnormalities in any cohort(Fig. 10B-C). Subsequently, liver and kidney functional integrity was assessed through standard serum toxicity markers. Serum alanine aminotransferase (ALT) and aspartate aminotransferase (AST) levels, indicative of hepatotoxicity, and blood urea nitrogen (BUN) and creatinine (CREA) levels, used for assessment of renal function, were all within normal ranges (Fig. 10D-G). The results indicated the attenuated engineered bacteria do not cause severe systemic toxic reactions, which is in comformity with our previous study [20].

## Discussion

The major findings of the present experiments can be summarized that (i) engineered Salmonella could cross BBB, targeted and colonized in infarcted area in dMCAO model mice; (ii) intravenous injection of engineered *Salmonella* (*S.t*-ΔpG^FGF21^) could continuously secret FGF21 with L-Arabinase induction and significantly improve neurological function recovery after IS; (iii) bacterial secreted FGF21 could effectively reduced the volume of cerebral infarction and inhibited neural apoptosis by up-regulation of autophagy through AMPK/mTOR signal pathway; (iv) intravenous injection of attenuated *Salmonella* do not cause severe global inflammatory reaction, or systemic toxic reactions in dMCAO model mice. These novel findings suggested that *Salmonella* could be used as a biosafe vehicle for delivering therapeutic proteins in stroke patients, providing a potential novel therapeutic tool for the clinical management of stroke.

Despite the rapid advances in modern medicine, many human diseases have so far lacked effective treatments. This has greatly facilitated the development of “unconventional” treatments such as viral, bacterial and plasmodial therapies [47]. William B. Coley used *Streptococcus* bacteria to treat inoperable clinical cancer patients more than 100 years ago [48]. It is now reported that the use of bacteria as carriers for the treatment of tumours such as pancreatic cancer [49], colon cancer [50], melanoma [51], glioma [20], etc. has shown significant therapeutic effects, and bacteria-based anticancer therapy can effectively break the limitations of current cancer therapies [52]. Moreover, bacterial therapy has an increasingly popular trend. Bacteria are able to target the necrotic areas of tumours and specifically multiply in the hypoxic and necrotic areas of tumours and achieve tumour suppression. *Salmonella* [53], *Escherichia coli* [54] and *Streptococcus* [55] are often used in tumour therapy. In particular, *Salmonella typhimurium*, which is a parthenogenetic anaerobic bacterium, has been studied more and is considered to be a therapeutic method with greater potential. For example, some of the more common engineered *Salmonella typhimurium* are VNP20009, A1-R, YB1 and SHJ2037. In addition, *Salmonella typhimurium* has been shown to target infarct foci in vivo, such as intestinal necrotic foci [56] and myocardial infarct foci [57]. Based on this property, we speculated whether *Salmonella* could target cerebral infarction foci. Based on our experimental results, we demonstrated that *Salmonella* can cross the blood-brain barrier and achieve specific targeting to cerebral infarct foci. This provides a new idea for clinical treatment of ischaemic stroke. Compared with viral vectors and bio-nanoparticles, the core advantages of bacterial vectors are targeted delivery capability, sustained protein expression and immunomodulatory synergy. *Salmonella* was found target hypoxic regions, while local inflammatory signal in infarcted aera attract accumulation of *Salmonella*. Engineered *Salmonella* can survive for days to weeks in lesions, enabling prolonged target protein (like FGF21) release, whereas viral vectors (e.g., adenovirus) suffer short-term expression ude to immune clearance. Also, bacterial components (e.g., LPS, flagellin) activate innate immunity, promoting reparative microglia (M2 polarization) [58], while viral vectors may trigger excessive inflammation. Viral vectors require direct intracranial injection (BBB bypass) [59], while bacteria can home to stroke sites after intravenously administration. Lentiviruses might pose genotoxicity risks, pre-existing immunity, and limiting redosing [13]. Similarly, nanoparticles exhibit low delivery efficiency (< 1% dose) due to partial BBB recovery post-stroke [16]. Compared with bacterial, passive targeting and limited payload restrict the application of lentiviruses and nanoparticles. Bacteria could proliferate localy while nanoparticles require repeated injections for continuous effect. As in potential applications, bacteria delivering CRISPR-Cas9 (VEGF editing) plus nanoparticle drug co-delivery might be future derections. Thus, the bacteria strategy exhibited advantage of biocompatibility, multifunctional engineering, and real-time monitoring (biolumines), which can greatly improve the therapeutic efficiency of the drug and reduce the systemic side effects of the drug. However, it is important to note that this bacterial delivery system also has its limitations. Future studies are needed to further optimize the system and address potential safety concerns, such as the possible over - growth of *Salmonella* in the body.

The mechanism by which bacteria target infarcted tissue is attractive. The speculation that the inflammatory environment may have attracted *Salmonella* is reasonable. Another possibility is that the infarcted area provides a metabolic environment favourable for anaerobic bacterial survival and proliferation [60]. The identification of inflammatory cell accumulation at the site of hypoxia and cytokine secretion by the infarcted tissue may provide clues to the mechanism by which *Salmonella* targets the infarcted area of the brain. Thus if their nature is further revealed, they could be used for other gene therapy or targeting strategies. In this study, we constructed a pBAD prokaryotic expression system that allows for controllable bacterial expression to regulate the timing and location of drug production in vivo. PBAD promoter transcriptional initiation can be controlled by interactions between AraC deterrents and L- Arabinose. We can modulate pBAD promoter-driven payload expression by intraperitoneal or intravenous injection of L-Arabinose [38, 53].

The BBB serves as a filtering barrier of capillaries, preventing most neural-protective agents from infarct aera and limiting their therapeutic effect, which contributes to the poor prognosis of stroke. *Salmonella typhimurium* can cross BBB and colonized in infarct aera, making it a potentially valuable drug carrier for treating stroke. Based on the above basis, we constructed *ΔppGpp* mutants by genetically engineering the endotoxin-associated pathogenicity genes relA and spoT in *Salmonellatyphimurium*, which resulted in the bacteria exhibiting weak virulence [53]. Using in vivo imaging system, we observed that the bacteria accumulate in the liver, spleen, kidneys, and other organs of mice within hours after tail vein injection and are subsequently gradually cleared by the immune system, with specific growth and multiplication lasting for about 2 weeks in the target zone only. In addition, we can deliberately terminate bacterial colonisation of infarcted tissues and eradicate bacterial residency in the body by artificially adding antibiotics (e.g. quinolones).

The brain is more delicate than other tissues, and bacterial infection can cause serious consequences. To further ensure biosecurity, we successively detected microglia activity and pro-inflammatory cytokines secretion post bacteria injection. We ovserved becteria injection could cause a certain elevation of global inflammatory level, and increased activation of microglia. However, this kind of amplification is acceptable, and causes no significant adverse impact in long-term recovery. We also monitored the body weight and hepatorenal function access bacterial toxicity, finding the body weight of the mice recovered rapidly after injection of the bacteria through tail vein. When examining the liver and kidney functions, we found that only the liver function indexes were transiently elevated and then gradually recovered to normal, while the kidney function was basically unaffected. These results indicates that engineered *Salmonella* has an acceptable biosafe profile.

Fibroblast growth factor (FGF) is a family of structurally related protein polypeptides that, together with their receptors, form a complex signalling system [61, 62]. In recent years, there has been increasing evidence that the FGF family plays a key role in the repair of central nervous system injury. FGF21’s multimodal mechanisms (neuroprotection, anti-inflammation, metabolism) and favorable safety profile make it superior to single-target growth factors (e.g., BDNF, VEGF) for ischemic stroke therapy [30]. Its endogenous regulatory role and clinical translatability further support its selection as a key therapeutic target [34]. FGF21 can inhibit neuronal apoptosis caused by hypoxic-ischemic brain injury in neonatal rats [25], protect and repair damaged neurons in a rat model of ischemia followed by reperfusion [63], attenuate disruption of the blood-brain barrier after cerebral infarction in diabetic mice, reduce the infarct size [26], and promote recovery of neurological function after MCAO in mice [27]. It was found that although neurons in the brain can express FGF21, its short half-life and poor stability limit its application [64, 65]. In contrast, many other neuroprotective factors may only have a single or limited set of functions [66, 67]. This makes FGF21 a more suitable candidate for promoting comprehensive neural repair after ischemic stroke. Therefore, we used engineered *Salmonella* carrying the FGF21 gene as a carrier, and by virtue of the bacteria’s ability to target and multiply in the brain infarct foci. We gave L-Arabinose to induce the bacteria to continuously produce and secrete FGF21 protein in large quantities. This not only greatly improves the therapeutic efficiency of FGF21, but more interestingly, we can artificially control the time and site of FGF21 expression by the bacterium, and we can achieve the continuous and stable expression of FGF21 by repeated induction.

FGF21 has been reported as a key mediator involving in AMPK/mTOR signaling pathway [68–70]. As a neuroprotective agent, FGF21 attenuated neural cell apoptosis, oxidative stress, and mitochondrial energy metabolism disorder [71, 72]. Several studies have indicated AMPK/mTOR signaling was related with autophagy [68, 73, 74] and played a critical role in ischemic stroke [68, 75]. p-AMPK inhibits the activation of the mTOR, which in turn promote autophagy degree to inhibit cell apoptosis. In this present study, administration of *S.t*-ΔpG^FGF21^ promote local expression of FGF21 around the infarct area, which stimulated phosphorylation of AMPK and hinder phosphorylation of mTOR. Western blot also showed decreased BAX expression and inactivated caspase 3 following induced *S.t*-ΔpG^FGF21^ treatment, along with elevated autophagy relative protein ATG5 and high ratio of LC3II/LC3I. These results suggested ischemia following dMCAO significantly provoked neuronal apoptosis, while *S.t*-ΔpG^FGF21^ injection dramatically promoted local high concentration of FGF21 and inhibited neuronal apoptosis by up-regulation of autophagy through AMPK/mTOR signal pathway, while the application of autophagy inhibitor 3-MA reverse this neuroprotective effect, indicating the pivotal role of autophagy in ischemia stroke induced neural apoptosis through FGF21/AMPK/ mTOR pathway.

In addition, we further investigated *Salmonella* colonisation of the infarcted area of the brain and its form of action in secreting the target protein. The results showed no obvious presence of *Salmonella* in vitro, along with no significant difference in the expression level of FGF21 in neuronal cells compared to the control group. This suggests that the bacteria do not function through entering into neuronal cells, nor do their secreted FGF21 intracellular. Combined with cellular and animal experiments, it was found that the level of FGFR1 phosphorylation was significantly higher in the bacterial-induced group, suggesting bacterially secreted FGF21 was functionally regulated by binding to the cell surface FGFR1 receptor.

It is important to note that *Salmonella* inevitably affects other types of neuronal cells in the brain. Microglia, due to their phagocytosis function, are activated when encountering factors such as bacterial infection, leading to the release of many pro-inflammatory factors and causing neuroinflammation [76, 77]. It is expected that a combination therapy with engineered bacteria carrying functional proteins that inhibit microglia M1 polarization may be able to suppress inflammation better, but since the bacteria themselves induce microglia M1 polarisation, further experiments are needed to investigate the results. Regarding the immunogenicity evaluation, the observation that bacterial injection initially exacerbated the infiltration of macrophages and T - cells in the brain is consistent with the fact that bacteria are foreign agents that can trigger an immune response. The immune system recognizes the bacterial components and activates immune cells to eliminate the invaders. The increased infiltration of macrophages and T - cells in the early stage may be part of the body’s natural defense mechanism. However, the fact that there was no significant difference between the dMCAO group and the bacteria - injected group on the 14th day implies that the immune response gradually subsided over time. This could be due to the clearance of bacteria by the immune system or the adaptation of the immune system to the presence of the bacteria.

We are aware of some limitations in this study. Firstly, the bacteria targeting the ischemic region of the brain can deliver therapeutic proteins to salvageable neuronal cells. However, neural cell are restrict with extracellular proteins. Beside these membrane receptor effective protein, lots of neuroprotective protein should enter intracellular to function, which limits its application and needs further design. Secondly, L-Arabinose was intraperitoneally injected to keep high blood level for bacterial protein induction. The side effects of L-Arabinose on the body need further testing. Meanwhile, induced *Salmonella* expressed a wide variety of bacterial inclusions, which may cause unexpected biological effects and body responses, requirement of further monitor and investigation [78–80]. Lastly, detailed molecular mechanisms for FGF21-induced AMPK actication and downstream signaling pathways in preventing neural apoptosis after dMCAO need to be elucidated in future investigations. Nevertheless, how the innate immune cells recruitment and activation, and their influence for this neuro-targeted therapy after engineered *Salmonella* infection need further attention.

## Conclusion

**Fig. 11:**
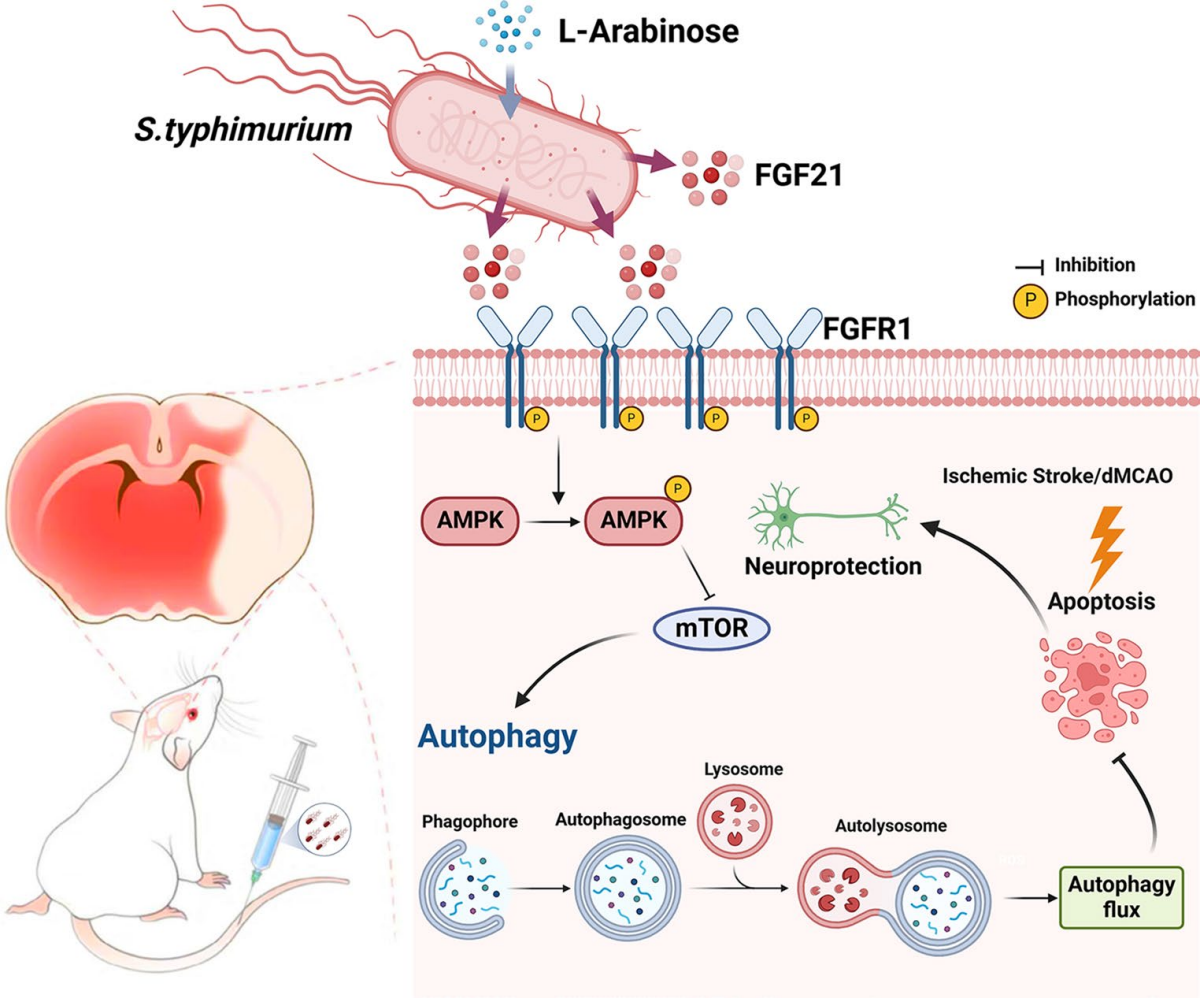
Schematic illustration of therapeutic mechanism of engineered *Salmonella* (*S.t*-ΔpG^FGF21^) in cerebral infarct foci

Overall, the present work firstly demonstrates engineered bacterial targeting and long-termly therapeutic efficacy for ischemic stroke in the world. This study verified bacterially secreted FGF21 reduced neural apoptosis after dMCAO by activating autophagy through its action on neuronal FGFR1 receptors, reduced infarct foci size, and improved neurological function in ischemic stroke mice (Fig. 11). Collectively, this study not only offers new insights into the neuroprotective effect of FGF21 but also presents a novel bacterial-based therapeutic strategy with infarct zone targeting and local long-term effect, which is far superior to traditional medicine therapy and is of great significance for the treatment of ischemic stroke.

## Electronic supplementary material

Below is the link to the electronic supplementary material.

Supplementary Material 1

## Data Availability

No datasets were generated or analysed during the current study.
