# Supplementary material for: Genetically-engineered Salmonella typhimurium expressing FGF21 promotes neurological recovery in ischemic stroke via FGFR1/AMPK/mTOR pathway
Source: J Neuroinflammation. 2025 Jun 28;22:170. doi: 10.1186/s12974-025-03498-0 (PMC12205506; doi:10.1186/s12974-025-03498-0)
Supplement: Supplementary file 1 — Supplementary Material 1 [file 12974_2025_3498_MOESM1_ESM.docx]

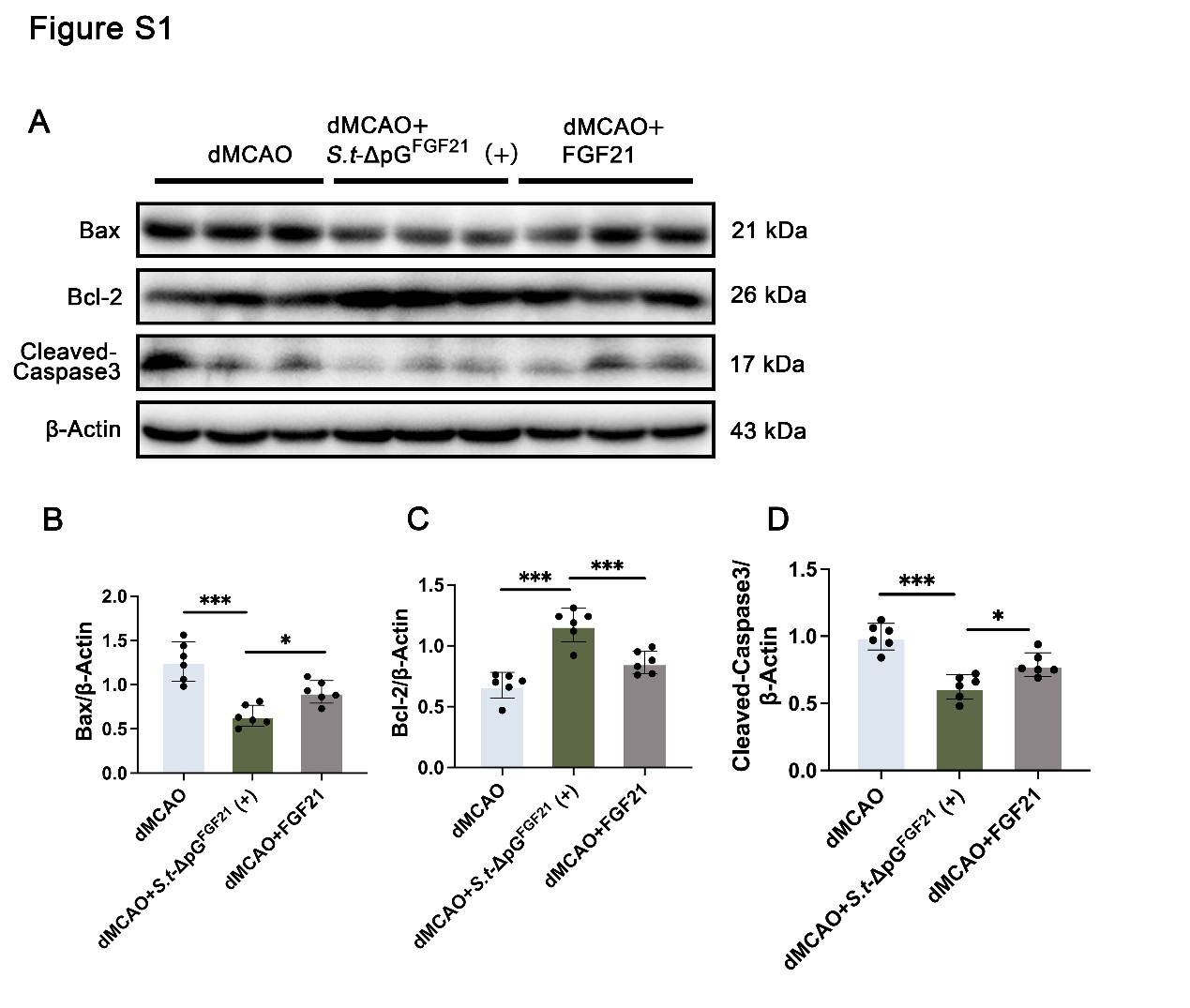


**Figure S1. *S.t*-ΔpG^FGF21^(+) superior to FGF21 single dose treatment in neuronal anti- apoptosis after stroke.** (A) Representative western blot images of Bax, Bcl-2, and Cleaved-Caspase3; (B)Relative quantitative analyses, n = 6. * *p* <0.05, *** *p* <0.001.


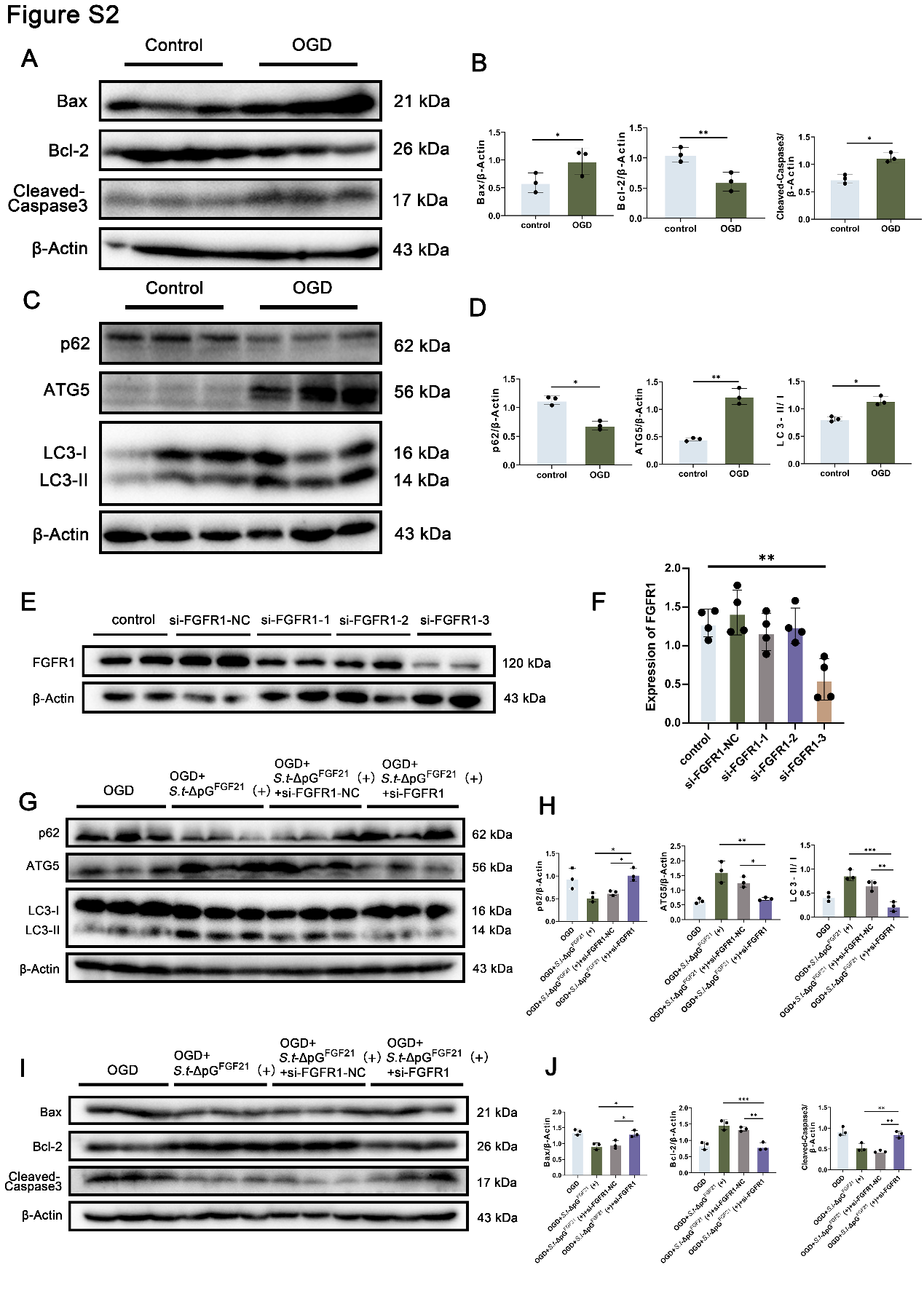


**Figure S2. FGFR1 Is Essential in FGF21-mediated pathway.** (A-B) Expression level and relative quantitative analysis of Bax, Bcl-2, and Cleaved-Caspase3 after OGD, n = 3; (C-D) Expression level and relative quantitative analysis of ATG5, P62, and LC3B after OGD, n = 3; (E-F) Determination of knockout efficiency of FGFR1 after siRNA-FGFR1 interference. (G-H) Expression level and relative quantitative analysis of Bax, Bcl-2, and Cleaved-Caspase3 after OGD, n = 3; (I-J) Expression level and relative quantitative analysis of ATG5, P62, and LC3B after OGD, n = 3. * p <0.05, ** p <0.01, *** p <0.001.


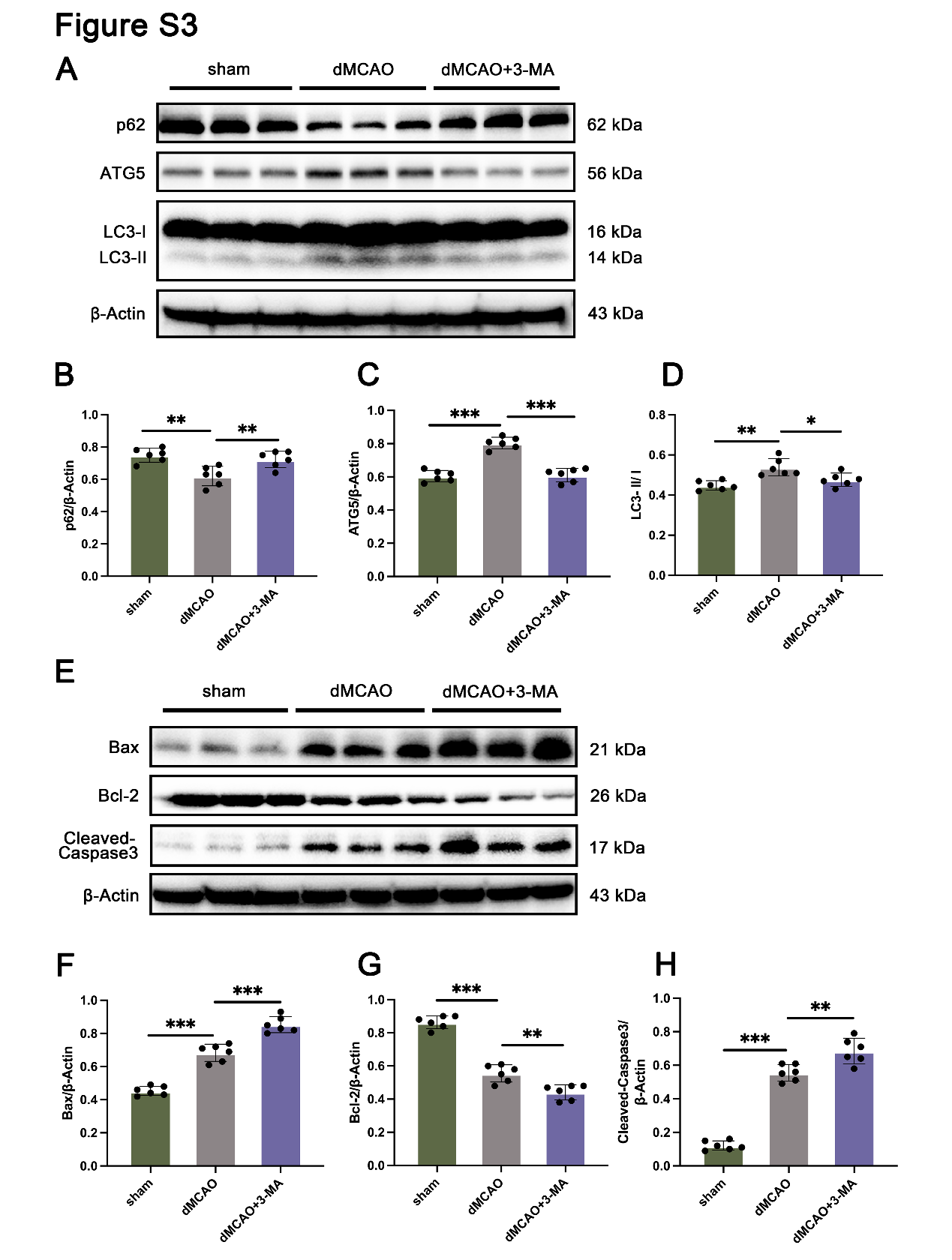


**Figure S3. Blockage of ischemia induced autophagy led to further cellular damage.** (A-D) Immunoblot detection of p62, ATG5, and LC 3 expression and relative quantification in the cerebral cortex of different groups, n = 6; (E-H) Expression level and relative quantification of apoptosis-related proteins Bax, Bcl-2, and Cleaved-Caspase3, n = 6. * p <0.05, ** p <0.01, *** p <0.001.
